# Hepatic stellate cells promote structural and functional remodeling of three-dimensional hepatocellular carcinoma coculture spheroids

**DOI:** 10.1007/s11033-026-12715-9

**Published:** 2026-09-12

**Authors:** Jessica Obelar, João G. Vasconcellos, Natália B. do Nascimento, Arieli C. de Sousa, Maria Giehl, Benjamim Simões Fischer, Janaína de Souza Ferreira, Jade de Oliveira, Vera M. T. Trindade, Fátima T. C. R. Guma

**Affiliations:** 1 Departamento de Bioquímica, Instituto de Ciências Básicas da Saúde, Universidade Federal Do Rio Grande Do Sul, Porto Alegre, Rio Grande Do Sul Brazil; 2 Programa de Pós-Graduação Em Ciências Biológicas: Bioquímica, Instituto de Ciências Básicas da Saúde, Universidade Federal Do Rio Grande Do Sul, Porto Alegre, Rio Grande Do Sul Brazil

**Keywords:** HCC, Spheroids, Tumor microenvironment, Cell death, Extracellular matrix, Sorafenib

## Abstract

**Background:**

Hepatic stellate cells (HSCs) are major regulators of the hepatocellular carcinoma (HCC) tumor microenvironment, contributing to extracellular matrix remodeling, tumor progression, and therapeutic resistance. Although three-dimensional (3D) coculture spheroid models better represent tumor-stroma interactions than conventional monocultures, the impact of HSCs on spheroid architecture and function remains incompletely characterized. This study investigated how HSCs influence the structural and functional properties of a 3D HCC model.

**Methods and results:**

Spheroids were produced using HepG2 cells, LX-2 cells (HSCs), or a 1:1 coculture system (CCS) and characterized over 96 h. Morphometric analysis demonstrated that CCS spheroids exhibited reduced area and perimeter together with increased circularity and normalized solidity, indicating enhanced structural organization and compaction. Scanning electron microscopy (SEM) confirmed distinct ultrastructural organization among the spheroid models. Flow cytometry revealed fewer membrane-compromised cells in CCS spheroids than in HepG2 spheroids. Although the percentage of active caspase−3-positive cells was unchanged, active caspase−3 fluorescence intensity was lower in both HepG2-like and LX-2-like populations. CCS spheroids showed higher collagen type I alpha-1-associated fluorescence, predominantly in the α-SMA-positive compartment, consistent with a stromal collagen-producing phenotype. Biochemical analyses revealed distinct extracellular lactate profiles. Extracellular LDH activity was lower in CCS spheroids than in HepG2 spheroids, although normalized LDH release did not differ between these groups. Functionally, CCS spheroids displayed reduced sorafenib sensitivity, with an IC30 approximately 3-fold higher than HepG2 monoculture.

**Conclusions:**

LX-2 incorporation was associated with greater spheroid organization, increased collagen type I-related expression, altered cell-death and extracellular metabolic profiles, and reduced sorafenib sensitivity, supporting the value of HepG2/LX-2 coculture spheroids for studying selected tumor–stromal interactions.

**Supplementary Information:**

The online version contains supplementary material available at https://doi.org/10.1007/s11033-026-12715-9.

## Highlights

- LX-2 incorporation altered the architecture and compaction dynamics of HepG2 spheroids.
- Coculture spheroids displayed greater circularity and a lower proportion of PI-positive cells than HepG2 spheroids.
- Collagen type I alpha-1-associated fluorescence was enriched in the α-SMA-positive compartment.
- Cellular composition altered active caspase-3 signal, extracellular LDH activity, and lactate accumulation.
- HepG2/LX-2 coculture spheroids required higher sorafenib concentration to achieve 30% inhibition.

**Supplementary Information:**

The online version contains supplementary material available at https://doi.org/10.1007/s11033-026-12715-9.

## Introduction

Hepatocellular carcinoma (HCC) is the most common primary liver cancer and ranks as the third leading cause of cancer-related death globally [1]. Despite advances in diagnosis and systemic therapies, clinical outcomes remain poor owing not only to the intrinsic heterogeneity of tumor cells, but also to the profound influence of the tumor microenvironment (TME) on tumor progression and therapeutic response [2]. Hepatic stellate cells (HSCs) are major components of the HCC microenvironment. In a healthy liver, HSCs remain quiescent and store vitamin A in intracellular lipid droplets [3]. Upon liver injury, they become activated, acquiring a myofibroblast-like phenotype and contributing to fibrogenesis through excessive extracellular matrix (ECM) secretion [3]. Persistent activation promotes fibrosis and can support tumor progression, invasion and therapeutic resistance [4]. Activated HSCs are also major producers of collagen type I (COL1) and other ECM proteins [5, 6]. Given their critical role, coculture models combining HCC cells and HSCs can provide a more physiologically relevant platform to study tumor behavior.

Traditionally, in vitro studies have relied on two-dimensional (2D) monocultures of HCC-derived cell lines such as HepG2. While useful, these models do not reproduce the structural and cellular complexity of tumors, particularly with respect to cell–cell and cell–matrix interactions [7]. As a result, 2D models often have limited predictive value for drug responses and tumor progression [8]. On the other hand, three-dimensional (3D) cell culture systems, such as spheroids, have gained prominence in cancer research because they reproduce selected spatial features of solid tumors, including oxygen, nutrient, and metabolite gradients. Compared with 2D monolayers, 3D cultures more accurately capture key biological features such as ECM organization, cellular polarity, and resistance to therapy [9]. 3D culture systems can also improve the predictive value of preclinical drug screening by more closely mimicking the TME [10]. However, studies that comprehensively investigate the morphometric characteristics of HCC–HSC coculture spheroids, including parameters such as shape, size, and compactness, remain limited.

Tumor morphology has emerged as a non-invasive indicator of biological behavior in imaging-based studies of several malignancies [11–14], including the prediction of microvascular invasion in HCC [15]. Compact and round shapes are frequently associated with less aggressive phenotypes, whereas irregular and lobulated structures correlate with increased malignancy [16]. Although shape analysis is already incorporated into computer-aided diagnosis (CAD) systems for breast and thyroid cancers in clinics [17], they support the broader premise that quantitative shape descriptors can carry biological information. Therefore, quantitative morphometric analysis may provide valuable descriptors of spheroid organization that can be integrated with functional assays to investigate how stromal cells influence tumor architecture, ECM remodeling, cell death, metabolic behavior, and therapeutic response. Integrating structural characterization with functional analyses may therefore provide a more comprehensive understanding of how HSCs regulate tumor biology in 3D HCC models.

In this study, we characterized 3D spheroids generated from HepG2 cells (HCC-derived), LX-2 (HSCs), and their CCS spheroids to investigate how stromal components influence tumor organization and function. Morphometric analysis was complemented by scanning electron microscopy (SEM), flow-cytometric assessment of cell-death-associated and collagen-related markers, biochemical assessment of membrane integrity and extracellular lactate, as well as functional determination of sorafenib sensitivity. By combining structural, molecular, and functional approaches, we aimed to determine how HSCs reshape spheroid architecture, COL1-related expression, cell death profiles, and therapeutic response in HCC. This integrated approach may contribute to the development of more physiologically relevant in vitro liver cancer models for mechanistic studies and preclinical drug screening.

## Methodology

### Cell culture

HepG2 cells were purchased from the Rio de Janeiro Cell Bank (#0103), and Dr. Karen C. Martinez de Moraes generously provided LX-2 cells from UNESP under the authorization of Prof. Scott Friedman. Both cell lines were maintained in low glucose (1 g/L glucose) Dulbecco’s Modified Eagle Medium (DMEM) (Sigma Aldrich, St. Louis, MO, USA) supplemented with 2% of fetal bovine serum (FBS) and 1% penicillin–streptomycin (final concentration of 100 units/mL penicillin and 100 µg/mL streptomycin) at 37 ºC in a humidified atmosphere containing 5% of CO₂. The cells were passaged using 0.25% trypsin–EDTA (Gibco, Grand Island, NY, USA).

### Spheroid establishment

**Fig. 1:**
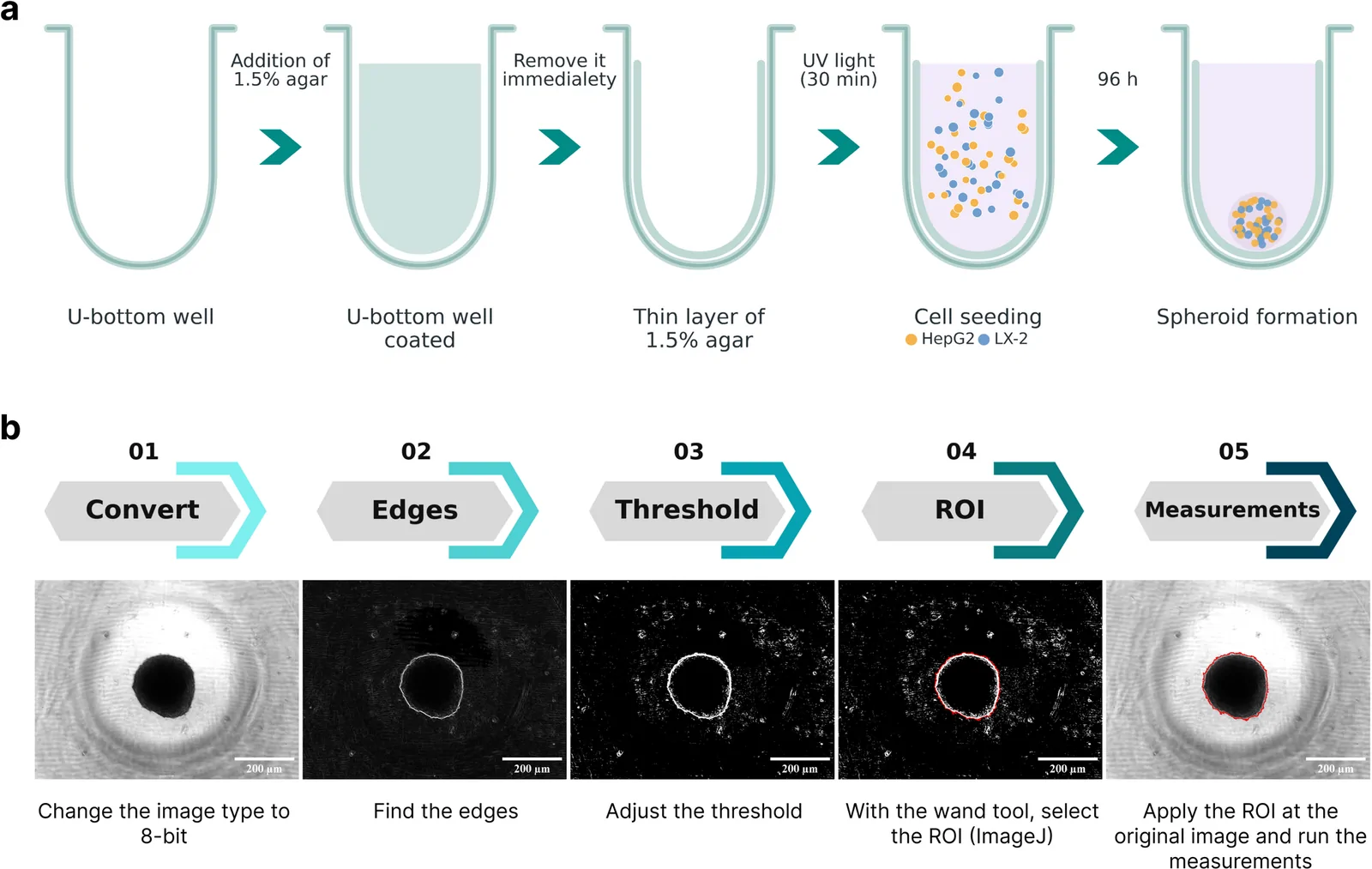
Scheme of how to prepare the plate for spheroid formation and morphometric evaluation. (**a**) Add 100 µL of Soft-agar 1.5% to each well using a multichannel pipette and immediately remove it from the wells. After 30 min under the UV light the wells come with a thin and sterile layer of 1.5% soft agar lining the surface. (**b**) Workflow for image processing and morphometric analysis in ImageJ: (01) convert the image to 8-bit; (02) detect edges; (03) adjust the threshold; (04) select the region of interest (ROI) using the wand tool; (05) apply the ROI to the original image and perform the measurements. Scale bar = 200 µm

96-well U-bottom plates (cat. no. 701111; Wuxi NEST Biotechnology Co., Ltd., Wuxi, Jiangsu, China) were coated with a thin layer of 1.5% (w/v) agar prepared in phosphate-buffered saline (1 × PBS) and sterilized by autoclaving. After the agar had solidified, the coated plates were exposed to UV light for 30 min [18]. HepG2 and LX-2 monoculture spheroids were generated by seeding 5 × 10^4^ cells per well (Fig. 1a). To model the cellular composition of the HCC-TME, LX-2 and HepG2 cells were co-seeded at a 1:1 ratio, with 2.5 × 10^4^ cells of each cell type per well, for a total density of 5 × 10^4^ cells/well, thereby establishing the CCS spheroids [19, 20].

### Optical microscopy

Spheroid morphology was monitored by phase-contrast microscopy using an Olympus IX70 inverted microscope equipped with a F-view digital camera. Images were acquired using a ×4 objective at 2, 24, 48, 72, and 96 h after cell seeding. The same spheroids were followed longitudinally by imaging the corresponding wells at each time point. This observational timeline spanned 96 h, enabling a thorough examination of the dynamic changes and growth patterns exhibited by the spheroids across their developmental stages.

### Morphometric analysis

To measure parameters such as area, density, aspect ratio, perimeter, circularity, roundness, and solidity in ImageJ (NIH, Bethesda, MD), a meticulous protocol was followed [21]. The image was converted to 8-bit, the scale was calibrated, and measurements were set to include area, mean gray value (MGV), centroid, perimeter, fit ellipse, shape descriptors, integrated density, limit to threshold, and display label. Subsequently, edges were defined, limits were adjusted, and a region of interest (ROI) was chosen using the ROI Manager. The process described above determines a ROI with specific coordinates for each sample. This coordinate (ROI) was then applied to the original image to measure previously defined parameters (Fig. 1B) [18].

#### Projected area

Projected spheroid area was quantified as the number of pixels within the ROI after spatial calibration [22]. Upon calibration of the spatial scale in the software, the area values were automatically converted from pixel counts to physical units (µm^2^) using the following equation:

1 $$Area=number\ of\ pixels \times (Pixel\ Width \times Pixel\ Height)$$

#### Circularity

Circularity was calculated to evaluate the contour regularity of the spheroids [23, 24]. This parameter integrates the area and perimeter of each object, providing a measure of how closely the shape approximates a perfect circle. Values range from 0 (extremely elongated or irregular shapes) to 1.0 (perfect circle). The formula used by the software is:

2 $$Circularity=(4\pi \times Area)/{Perimeter}^{2}$$

#### Roundness

Roundness was assessed to determine the degree of elongation of the spheroids, independently of contour irregularities. Values near to 1 indicate a nonelongated fitted ellipse, whereas greater values suggest structural anisotropy or elongation [24, 25]. This descriptor considers the ratio between the area and the square of the major axis length, and was calculated using the following formula:

3 $$Roundness=(4\times Area)/(\pi \times {Major\ axis}^{2})$$

#### Aspect ratio

The aspect ratio of each spheroid was calculated from the major and minor axis measurements generated by ImageJ, which are based on an ellipse fitted to the selected ROI. When its value is 1, this indicates a symmetrical profile, while deviations reflect elongated or irregular morphologies [18, 25]. The aspect ratio was determined using the following formula:

4 $$Aspect\ ratio=(Major\ axis)/(Minor\ axis)$$

#### Solidity

Two different approaches were employed to assess solidity in spheroids: geometric solidity, based on shape descriptors; and normalized solidity, derived from grayscale intensity measurements [24, 26, 27]. Geometric solidity is a descriptor of morphological compactness. It quantifies how closely the spheroid’s shape approximates that of a perfect convex structure. Values close to 1 indicate a smooth, compact shape with regular edges, while lower values suggest irregularity, fragmentation, or surface roughness [28]. This metric was calculated in ImageJ using the formula:

5 $$Geometric\ solidity=Area/(Convex\ area)$$

To complement geometric morphometric parameters, spheroid optical appearance was accessed from bright-field images using MGV. Under standardized image-acquisition and processing conditions, lower MGV values indicated darker and less translucent spheroid images. Because geometric solidity reflects only the shape of the segmented object and does not incorporate pixel intensity, a study-specific MGV-derived optical compaction index (MGVi) was calculated by reversing and rescaling the MGV values as follows

6 *MGVi=(MGVmax-Current\ MGV)/(MGVmax-MGVmin)*

where MGVi represents the MGV of the individual spheroid, and MGVmax and MGVmin represent the maximum and minimum MGV values observed within each independent biological experiment. The transformation generated a dimensionless index ranging from 0 to 1, in which higher values corresponded to lower MGV and therefore to a darker, less translucent spheroid image. This index was interpreted as an indirect measure of apparent optical compaction rather than as a direct measurement of cellular density, ECM content, or mechanical stiffness.

#### Mean gray value/optical density

Spheroid grayscale intensity was assessed using the MGV, a parameter provided by ImageJ that reflects the average pixel intensity within a defined ROI. This measurement provides a straightforward quantitative estimate of grayscale density in brightfield images. Lower MGV corresponds to darker regions and may be indicative of increased biological density, such as greater cellular compaction, ECM accumulation, or necrotic areas. Conversely, larger MGV suggests brighter, less dense regions, potentially associated with reduced cell packing or looser tissue organization. This parameter was interpreted as a relative measure of spheroid grayscale intensity rather than as a direct measurement of cellular intensity of cellular density or compaction, because pixel intensity may also be influenced by spheroid thickness, internal cell distribution, ECM accumulation, and image-acquisition conditions [29]. It was calculated using the following formula:

7 $$Mean\;Gray\;Value = (\sum P ixel\;\operatorname{int} ensity)/({n^{\underline \circ }}of\ pixels\;in\;the\;ROI)$$

The equivalent circular diameter was subsequently estimated from the measured projected area by assuming a circular 2D profile. The complete calculation is provided in Supplementary Methodology S1.

### Scanning electron microscopy (SEM)

After 96 h of culture, spheroids were transferred to microcentrifuge tubes, washed once with 0.2 M sodium phosphate buffer, and fixed in 2.5% (v/v) glutaraldehyde prepared in the same buffer for one week at 4º C. Samples were then washed three times with sodium phosphate buffer, rinsed with distilled water, and dehydrated through a graded ethanol series (30, 50, 70, 90, and 100% for 10 min each). Following critical-point drying with a BALZERS CPD030 system, the spheroids were mounted, sputter-coated with gold, and examined using a Zeiss EVO MA10 SEM at 10 kV and a working distance of 8.5, 9 and 9.5 mm.

### Annexin V-FITC/PI

To characterize the cell-death profile of the spheroid models, Annexin V-FITC/propidium iodide (PI) staining was performed. For each biological replicate, ten spheroids from each group were collected, centrifuged at 1,200 rpm for 10 min, washed once with 1× PBS, and dissociated with 300 µL of 0.25% Trypsin–EDTA. The resulting cell suspension was centrifuged, washed once with 1 × PBS, and incubated with Annexin V-FITC (4 µg/mL, QuatroG) and PI (40 µg/mL, Invitrogen) for 15 min at room temperature [30, 31]. Following staining, the cells were washed and resuspended in 300 µL of 1 × Annexin V binding buffer. Data were acquired using a FACS Calibur flow cytometer (BD Biosciences San Jose, CA, USA), with 10,000 events collected per sample. Unstained controls were used to establish quadrant boundaries. During analysis, cellular events were first selected based on forward-scatter and side-scatter characteristics to exclude debris. Cell populations were classified as viable (Annexin V−/PI−; Q4), early apoptotic (Annexin V+/PI−; Q3), late apoptotic (Annexin V+/PI+; Q2), or PI-positive only (Annexin V−/PI+; Q1). The total PI-positive population, used as an indicator of plasma membrane compromise, was calculated as PI+ positive cells. Data was analyzed using FlowJo® version 10 (FlowJo LLC, Ashland, OR, USA).

### Determination of intracellular and extracellular lactate dehydrogenase (LDH) activity

Intracellular and extracellular lactate dehydrogenase (LDH) activities were quantified in HepG2, LX-2, and CCS spheroids after 96 h of culture as biochemical indicators of plasma membrane integrity [32]. For each biological replicate, four spheroids were pooled. The entire culture supernatant, corresponding to a total extracellular volume of ~ 350 μL, was collected and centrifuged to remove cellular debris and cells in suspension. An aliquot of 10 μL of the clarified supernatant was used for extracellular LDH measurement. For intracellular LDH determination, pooled spheroids were washed with PBS and lysed in a total volume of 100 μL of 1% Triton X-100 for 10 min at room temperature. The lysates were subsequently collected and used for intracellular LDH measurements.

LDH activity was measured using a commercial enzymatic kit (VIDA Biotecnologia, Brazil) according to the manufacturer’s instructions. NADH oxidation was monitored kinetically at 340 nm using a SpectraMax M5 microplate reader (Molecular Devices, USA) maintained at 37 °C. The rate of NADH oxidation was determined from the change in absorbance over time (ΔA/min) and converted to enzyme activity (U/L) according to the manufacturer’s recommendations. Three independent biological replicates were analyzed for each experimental condition. To estimate membrane damage, the percentage of LDH release was calculated as:

8 $$\mathrm{LDH\ Release\ (\%)}=\frac{\mathrm{Extracellular\ LDH\ (U/L)}}{\mathrm{Extracellular\ LDH\ (U/L)}+\mathrm{Intracellular\ LDH\ (U/L)}}\times100$$

### Determination of extracellular lactate levels

Extracellular lactate concentration was measured in conditioned medium from HepG2, LX-2, and CCS spheroids after 96 h of culture [33]. For each biological replicate, four spheroids were pooled, corresponding to a total conditioned-medium volume of 240 μL. The culture medium was collected and centrifuged at 1000 rpm for 5 min to remove cellular debris and suspended cells. The clarified supernatant, corresponding to the same extracellular fraction used for LDH activity measurements, was subsequently used for lactate determination.

Lactate concentration was measured using a commercial enzymatic kit (VIDA Biotecnologia, Brazil) according to the manufacturer’s instructions. Briefly, 2 μL of clarified conditioned medium was incubated with 200 μL of the reaction mixture, and absorbance was measured at 546 nm using a SpectraMax M5 microplate reader (Molecular Devices, USA). Lactate concentrations were calculated from a standard curve prepared using known lactate concentrations and expressed as mg/dL. A cell-free culture-medium control incubated under the same experimental conditions was used to account for baseline lactate present in the medium and background absorbance. The blank-corrected lactate concentration was calculated by subtracting the value measured in the cell-free medium control from that measured in the corresponding conditioned medium. Three independent biological replicates were analyzed for each spheroid model. Because lactate values were not normalized to cell number, DNA, protein content, or spheroid biomass, the results are reported as lactate concentration in conditioned medium rather than as a cell-specific lactate production rate.

### Flow cytometric immunofluorescence analysis

The intracellular abundance of hepatocyte nuclear factor 4 alpha (HNF4α), active caspase−3, alpha-smooth muscle actin (α-SMA), and collagen type I alpha 1 (COL1A1) was evaluated by flow cytometry using two separate staining panels: HNF4α/active caspase−3 and α-SMA/COL1A1. Detailed information on the antibodies used, including manufacturers, catalog numbers, fluorophores, and working dilutions, is provided in Supplementary Table S1. For each biological replicate, 6–8 spheroids per group were pooled after 96 h of culture and dissociated into single-cell suspensions using TrypLE Express for 10 min at room temperature, followed by gentle mechanical dissociation. Cells were fixed and permeabilized using the BD Cytofix/Cytoperm™ kit (BD Biosciences, San Jose, CA, USA; Cat. No. 554714) for 20 min at 4 °C. Primary and secondary antibodies were diluted in BD Perm/Wash buffer and incubated for 30 min at 4 °C protected from light. Samples were washed twice after each antibody-incubation step and finally resuspended in 100 µL of PBS. Unstained, single-stained, and secondary-antibody-only controls were included for gate definition, fluorescence compensation, and evaluation of nonspecific secondary-antibody fluorescence. Data were acquired using a BD Accuri™ C6 flow cytometer (BD Biosciences, San Jose, CA, USA), with 10,000 events collected per sample. Cellular events were selected using FSC-A/SSC-A gating, followed by singlet discrimination using FSC-H versus FSC-A. The percentage of positive cells and median fluorescence intensity (MFI) were determined using FlowJo® version 10 (FlowJo LLC, Ashland, OR, USA). Corrected MFI was calculated by subtracting the MFI of the corresponding matched negative control from that of the stained sample. Detailed antibody information and staining conditions are provided in the Supplementary Methodology S2.

### Evaluation of spheroid viability

After 48 h of treatment with sorafenib (Sigma, Cat. No. SML2633) at concentrations of 0–50 μM, cell viability was evaluated using the CellTiter-Glo® 3D Cell Viability Assay (Promega Corporation, Madison, WI, USA, Cat. No. G9681), which quantifies ATP as an indicator of metabolically active cells. Prior to the assay, the culture plates and the reagent were equilibrated to room temperature for approximately 30 min. Next, the volume of reagent added to each well was in a 1:1 ratio to the volume of medium. To ensure complete lysis of the 3D structures, we rapidly homogenized the wells using a multichannel pipette. Following this step, the plate was incubated for an additional 25 min at room temperature and in the dark, allowing the signal to stabilize. Luminescence was quantified in a SpectraMax M5 microplate reader (Molecular Devices, USA). Three independent biological experiments were performed, with four technical replicate spheroids per condition in each experiment. The results were then used to calculate the spheroid IC30 values [34].

### Statistical analysis

Data were analyzed using GraphPad Prism version 9.0.0 (GraphPad Software, San Diego, CA, USA). Morphometric parameters were analyzed by two-way analysis of variance (ANOVA), considering spheroid type and culture time as independent factors, followed by Tukey multiple comparisons test. Temporal changes within each spheroid model were further evaluated by one-way ANOVA followed by Sidak multiple comparisons test. Morphometric data are expressed by mean ± standard error of the mean (S.E.M). Cell death determined by Annexin V-FITC/PI assay, intracellular and extracellular lactate dehydrogenase (LDH) activity, extracellular lactate concentration, and COL1A1 expression among spheroid models were analyzed by one‑way ANOVA followed by Tukey’s multiple comparisons test. Active caspase−3 expression was analyzed by one-way ANOVA followed by Tukey’s multiple comparisons test for comparisons among spheroid models and by Šídák’s multiple comparisons test for comparisons between HepG2 and LX-2 monocultures and their corresponding HepG2-like and LX-2-like populations within CCS spheroids. Comparisons of α-SMA expression and COL1A1 expression between HepG2-like and LX-2-like populations within CCS spheroids were performed using an unpaired Student’s t-test. Data from biological replicates are presented as the mean ± standard deviation (SD).

## Results

### Morphometric dynamics

The LX-2, HepG2, and 1:1 LX-2/HepG2 coculture spheroids were generated (Fig. 1a) and monitored by phase-contrast microscopy for 96 h (Fig. 1b).

**Fig. 2:**
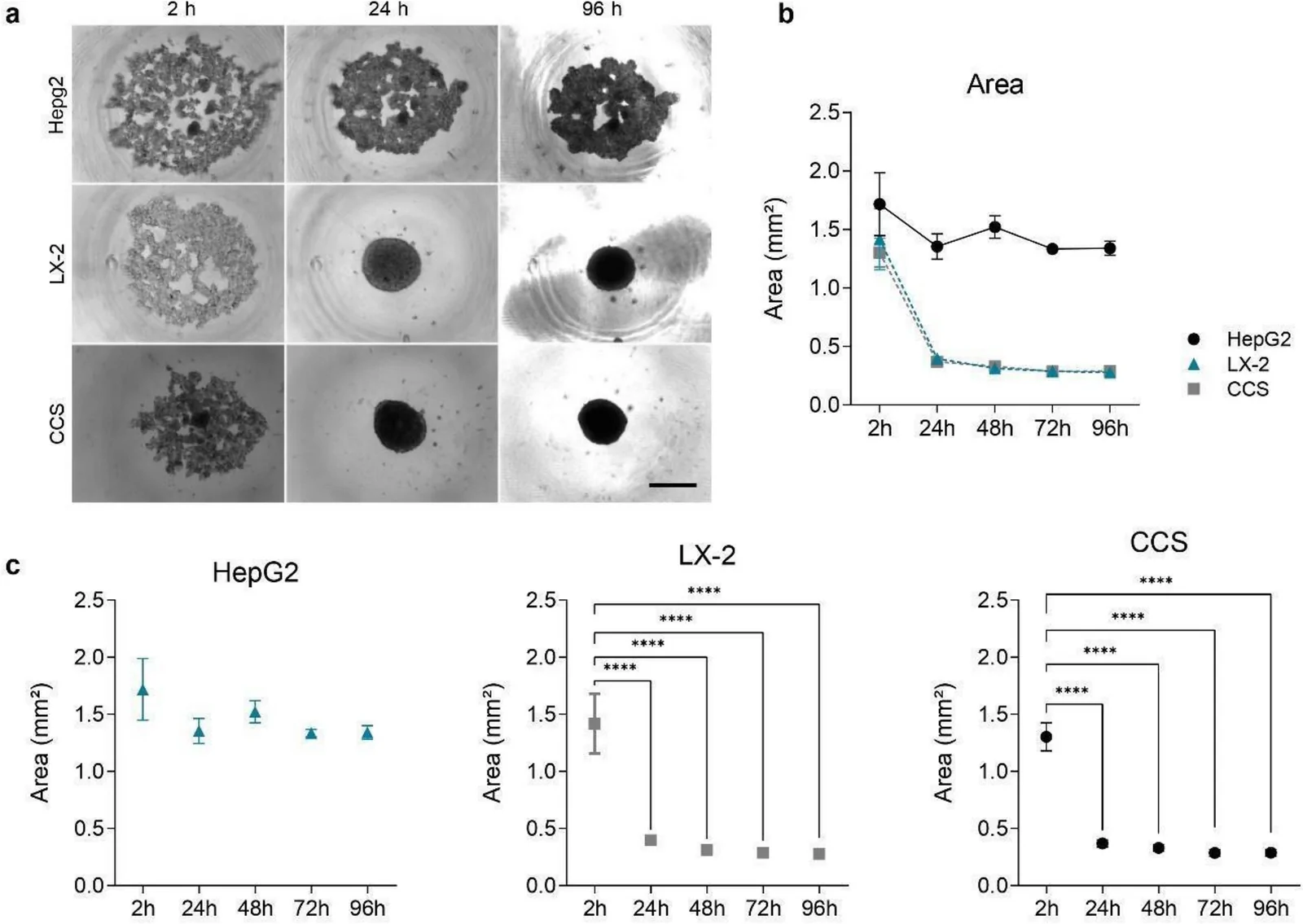
Time-dependent changes in the projected area of HepG2, LX-2, and CCS spheroids. (**a**) Representative phase-contrast images of HepG2, LX-2, and CCS spheroids after 2, 24, and 96 h of culture. Scale bar: XX µm. (**b**) Projected spheroid area measured at 2, 24, 48, 72, and 96 h, showing the temporal profiles of HepG2, LX-2, and CCS spheroids. (**c**) Time-dependent changes in projected area analyzed separately within each spheroid model. Data are presented as mean ± SEM. Data were analyzed by two-way ANOVA, which identified significant effects of spheroid type, culture time, and their interaction, followed by Šídák’s multiple-comparisons test. ****p < 0.0001

Although the same number of cells was initially seeded in each group, the projected area evolved differently among the spheroid models. Two-way ANOVA showed significant effects of spheroid type (p<0.0001), culture time (p<0.0001), and their interaction (p=0.0105; Fig. 2a, b). At 2 h, HepG2 spheroids already exhibited a larger projected area than LX-2 and CCS spheroids (Fig. 2a, b). These differences became more pronounced over time, and at 96 h the projected area of HepG2 spheroids was 4.8-fold greater than that of LX-2 spheroids and 4.6-fold greater than that of CCS spheroids (both *p* < 0.0001; Fig. 2b; Supplementary Table S2). The projected area of HepG2 spheroid decreased by 21.9% between 2 and 96 h, from 1.718 ± 0.27 mm^2^ to 1.341 ± 0.06 mm^2^, although this change was not statistically significant (*p* = 0.1002; Fig. 2c; Supplementary Table S3). In contrast, both LX-2 and CCS spheroids underwent marked compaction within the first 24 h, with reduction of projected area of 72% and 71.7%, respectively (both *p* < 0.0001, Fig. 2c; Table S3). By 96 h, the projected area of the LX-2 spheroids had decreased by 80.42%, from 1.419 ± 0.26 mm^2^ to 0.2778 ± 0.02 mm^2^ (*p* < 0.0001). CCS spheroids showed a similar reduction of 77.9%, decreasing from 1.303 ± 0.12 mm^2^ at 2 h to 0.2882 ± 0.03 mm^2^ at 96 h (*p* < 0, 0001, Fig. 2c; Supplementary Table S3).

**Fig. 3:**
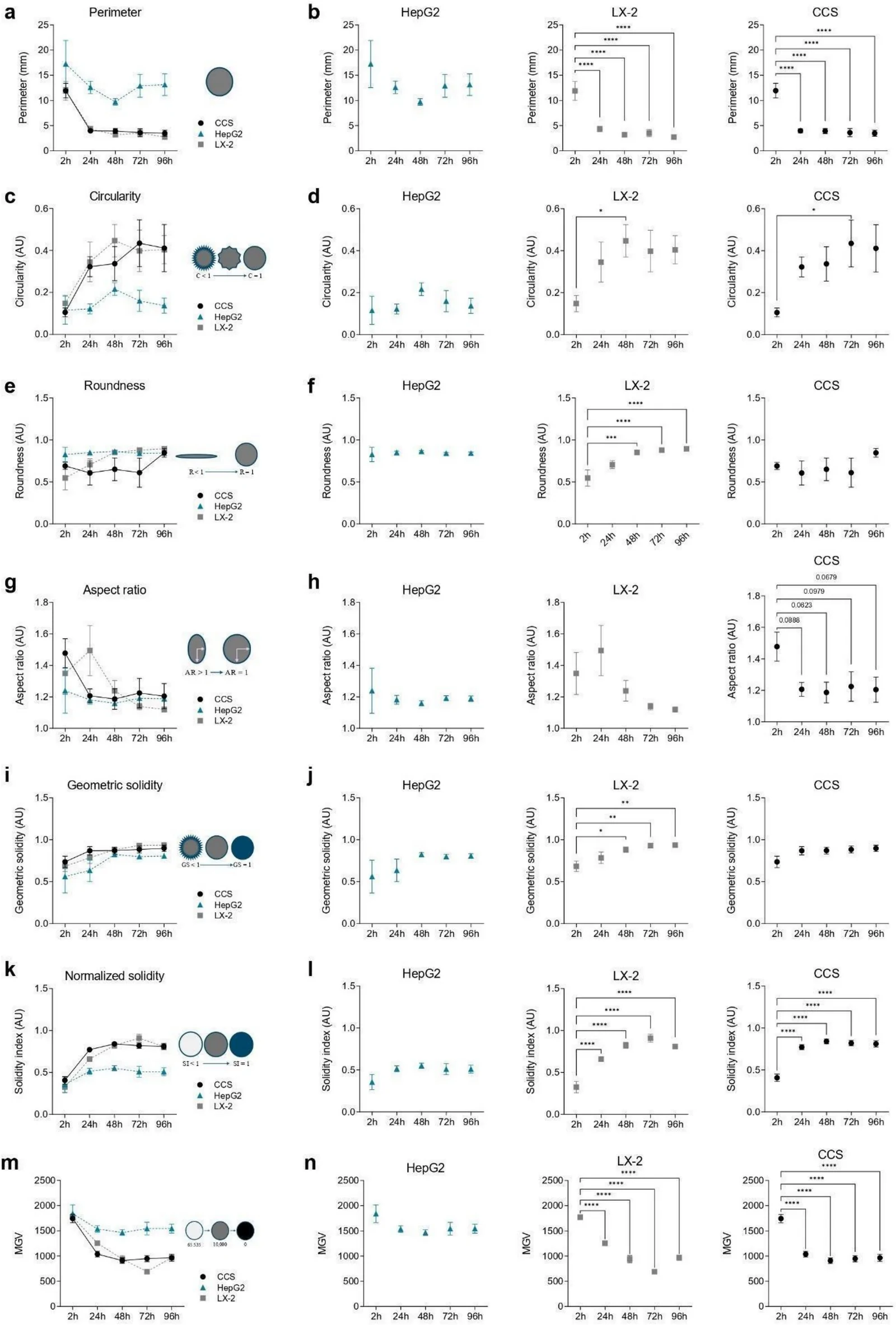
Morphometric properties across time points in HepG2, LX-2, and CCS cell lines cultures in 3D. (**a-m**) Quantification of various morphological properties of HepG2 (blue), LX-2 (gray), and CCS (black) cells at different time points (2 h, 24 h, 48 h, 72 h, 96 h). Panel (**a-b**) shows perimeter (mm), (**c-d**) circularity, (**e–f**) roundness, (**g-h**) aspect ratio, (**i-j**) geometric solidity, (**k-l**) normalized solidity, and (**m–n**) MGV. Significant differences are indicated by statistical annotations, with *p < 0.05, **p < 0.01, ***p < 0.001, and ****p < 0.0001. Data are presented as mean ± standard error of mean

A similar time-dependent pattern was observed for spheroid perimeter. This parameter was influenced by both culture time (*p* < 0.0001) and spheroid type (*p* < 0.0001); however, no significant interaction between these factors was detected (*p* = 0.6893; Fig. 3a). LX-2 and CCS spheroids exhibited similar temporal profiles, with marked reductions in perimeter during the first 24 h of culture: 63.6% for LX-2 and 66.9% for CCS spheroids (both *p* < 0.0001; Supplementary Table S4). Thereafter, perimeter values remained relatively stable, without further pronounced changes (Fig. 3a). Conversely, HepG2 spheroids showed a smaller reduction in perimeter over time. Between 2 and 96 h, the perimeter of HepG2 spheroids decreased by 23.8%, although this observation was not statistically significant (p = 0.4939; Fig. 3b; Supplementary Table S4). Compared with HepG2 spheroids, LX-2 spheroids exhibited a significant smaller perimeter at 24 h (*p* < 0.0001), 48 h (*p* = 0.0020), 72 h (*p* < 0.0001), and 96 h (*p* < 0.0001; Supplementary Table S5). Similarly, CCS spheroids showed a smaller perimeter then HepG2 spheroid at 24 h (*p* < 0.0001), 48 h (*p* = 0.0068), 72 h (*p* < 0.0001), and 96 h (*p* < 0.0001; Supplementary Table S5). By 96 h, the perimeter of LX-2 and CCS spheroids had decreased by 77.0% and 70.8%, respectively, relative to the 2-h values (both *p* < 0.0001; Fig. 3b).

In contrast to the early changes detected in area and perimeter, differences in circularity among the spheroid models emerged only at later culture times. Circularity was influenced by culture time (p = 0.0087) and spheroid type (p = 0.0001), whereas no significant interaction between these factors was detected (*p* = 0.7376). No differences among the spheroid models were observed up to 48 h of culture. At 72 h, HepG2 spheroids exhibited lower circularity than CCS spheroids (*p* = 0.0210; Supplementary Table S6). At 96 h, HepG2 spheroids were less circular than both LX-2 (*p* = 0.0256) and CCS spheroids (*p* = 0.0215; Fig. 3c; Supplementary Table S6). At the final time point, the mean circularity values were 0.1373 for HepG2, 0.4045 for LX-2, and 0.4112 for CCS spheroids (Fig. 3d; Supplementary Table S7). However, within-model comparisons between 2 and 96 h did not detect statistical changes in circularity for HepG2 (*p* = 0.9858), LX-2 (*p* = 0.0927), or CCS spheroid (*p* = 0.0706).

Roundness evolved differently among the three spheroid models, with the most pronounced changes occurring in LX-2 spheroids. Two-way ANOVA identified a significant effect of spheroid type (*p* = 0.0150), whereas the effect of culture time and the spheroid type × time interaction did not present any significant differences (Fig. 3e). Despite the limited overall change observed in HepG2 spheroids, their mean roundness increased slightly from 0.8267 at 2 h to 0.8425 at 96 h, corresponding to an increase of 1.9%, which is not statistically significant (*p* = 0.9733; Fig. 3f; Supplementary Table S8). In contrast, LX-2 spheroids exhibited a marked increase in roundness over time. Their mean roundness was 0.547 at 2 h and increased to 0.8514 at 48 h (*p* = 0.0005), 0.8797 at 72 h (*p* < 0.0001), and 0.8940 at 96 h (*p* < 0.0001) relative to the 2-h value. This represented an overall increase of 63.5% by the end of the culture period (Fig. 3f; Supplementary Table S8). CCS spheroids showed a more moderate increase, from 0.6900 at 2 h to 0.8473 at 96 h. Although this corresponded to an increase of 22.8%, the difference between the two time points was not statistically significant (*p* = 0.7547; Fig. 3f; Supplementary Table S9).

A different pattern emerged for aspect ratio, for which culture time was the only significant factor identified by two-way ANOVA (*p* = 0.0255). Neither spheroid type (*p* = 0.2564) nor the spheroid type × time interaction (*p* = 0.1013) significantly affected this parameter (Fig. 3g). Despite the significant overall effect of time, multiple-comparisons showed that the aspect ratio at 96 h did not differ significantly from the corresponding 2-h value in any spheroid mode (Supplementary Table S10). Between 2 and 96 h, aspect ratio decreased by 4.1% in HepG2, 17.0% in LX-2, and 18.5% in CCS spheroids, respectively, although none of these individual changes reached statistical significance (Fig. 3h; Supplementary Table S11).

Geometric solidity remained relatively stable in HepG2 and CCS spheroids, whereas LX-2 spheroids exhibited a progressive increase at later culture times. Two-way ANOVA showed that spheroid type, culture time and the spheroid type × time interaction were not significant (Fig. 3i). At 24 h, HepG2 spheroids exhibited 26.8% lower geometric solidity than CCS spheroids (*p* = 0.0303; Supplementary Table S12). Between 2 and 96 h, geometric solidity increased by 43.7% in HepG2 spheroids and by 21.9% in HepG2 spheroids, however, neither change was statistically significant (*p* = 0.23640 and *p* = 0.08940, respectively; Fig. 3j; Supplementary Table S13). On the other hand, LX-2 spheroids showed a significant increase in geometric solidity relative to the 2-h value, with increases of 28.935% at 48 h (*p* = 0.02050), and 36.782% at 96 h (*p* = 0.00450; Fig. 3j; Supplementary Table S13).

Optical compaction, accessed using the MGV-derived compaction index, changed markedly during spheroid formation. Two-way ANOVA identified significant effects of culture time (*p* < 0.0001), spheroid type (*p*< 0.0001), and their interaction (*p* = 0.0029) on this parameter (Fig. 3k). At 2 h, the compaction index did not differ among the spheroid models (Supplementary Table S14). By 24 h, CCS and LX-2 spheroids exhibited index values 33.407% and 28.330% higher than HepG2 spheroids, respectively (*p* = 0.0001 and *p* = 0.0421). At 96 h, the index remained higher in CCS and LX-2 spheroids than in HepG2 spheroids, by 37.1% and 58.7%, respectively (both *p* < 0.0001; Fig. 3k; Supplementary Table S14). Within each model, the HepG2 spheroid compaction index increased by 43.4% between 2 and 96 h, although this change was not statistically significant (*p* = 0.19480; Fig. 3l; Supplementary Table S15). In contrast, LX-2 spheroid showed increases of 102.6% at 24 h 148.4% at 96 h relative to the 2-h value (both *p* < 0,0001). CCS spheroids followed a similar pattern, with increases of 90.1% at 24 h and 99.6% at 96 h (both *p* < 0.0001; Fig. 3l).

Changes in optical appearance were reflected by MGV, with lower values indicating darker images and greater apparent optical density under standardized acquisition conditions. Two-way ANOVA identified significant effects of culture time (*p* < 0.0001), spheroid type (*p* < 0.0001, Supplementary Table S16), and their interaction (p = 0.0004) on MGV (Fig. 3m). At 2 h, MGV did not differ among the spheroid models. By 24 h, HepG2 spheroids exhibited an MGV 18.3% higher than LX-2 spheroids (*p* = 0.0176) and 47,834% higher than CCS spheroids (*p* < 0.0001; Supplementary Table S17). This general pattern was maintained at later culture times. At 72 h, CCS spheroids exhibited a 27.2% higher MGV than LX-2 spheroids (*p* = 0.0427). At 96 h, HepG2 spheroids retained higher MGV values than both LX-2 and CCS spheroids, by 37.3% and 60.1%, respectively (both *p* < 0.0001; Fig. 3m). Within-model comparisons showed no significant change in HepG2 MGV over time. In contrast, MGV decreased in LX-2 spheroids by 29.2% at 24 h and 45.4% at 96 h relative to the 2-h value (both *p*< 0.0001). CCS spheroids showed a similar reduction, with MGV decreasing by 40.4% at 24 h and by 44.7% at 96 h (both *p* < 0.0001; Fig. 3n). These reductions indicated that LX-2 and CCS spheroids became progressively darker during culture.

### Ultrastructural characterization of spheroid morphology

**Fig. 4:**
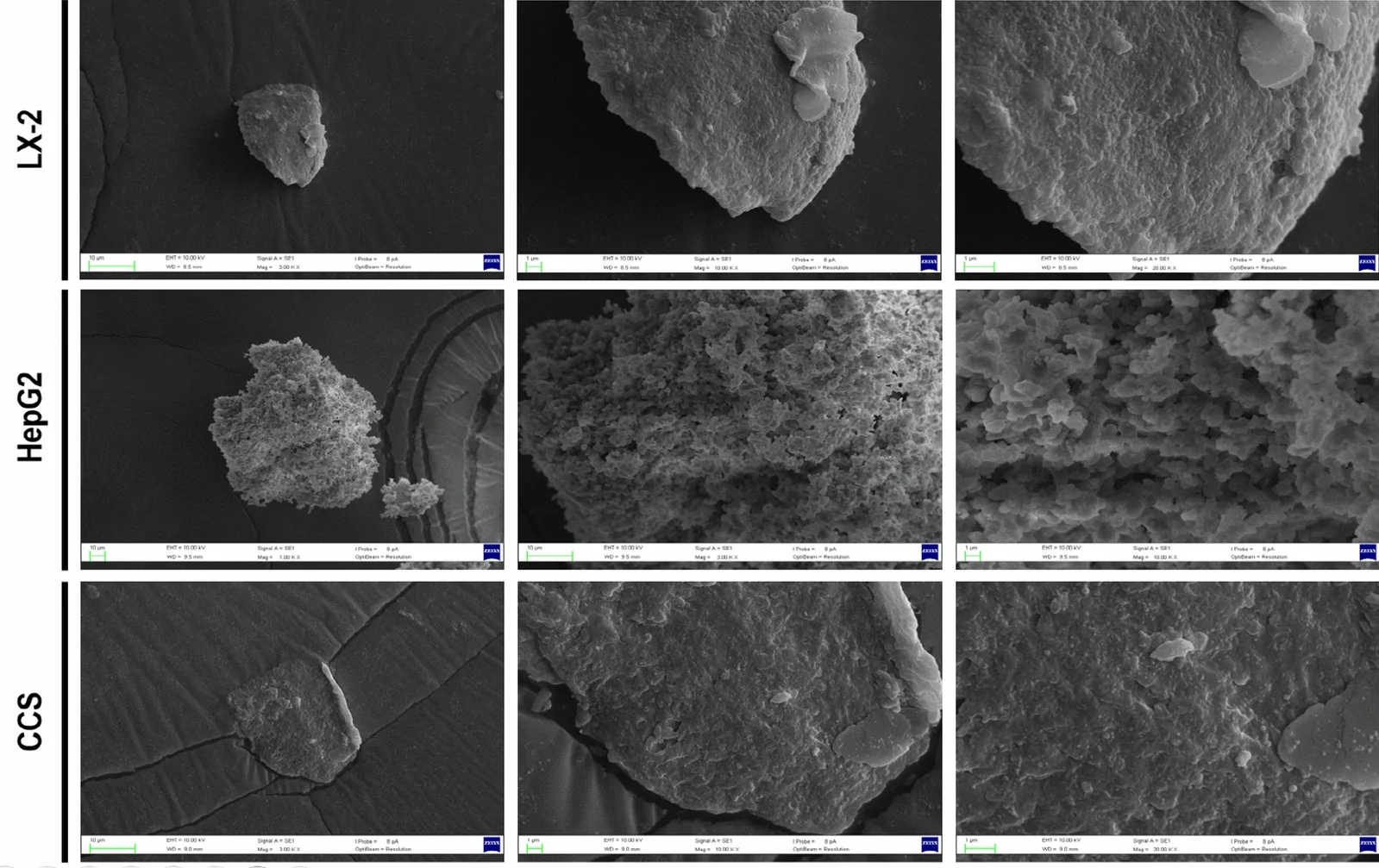
Ultrastructural characterization of LX-2, HepG2, and CCS spheroids by scanning electron microscopy (SEM). Representative SEM images acquired after 96 h of spheroid culture. LX-2 spheroids (top row), HepG2 spheroids (middle row), and CCS spheroids (bottom row) are shown at increasing magnifications to visualize overall spheroid morphology and surface ultrastructure. Images were obtained at 1,000×, 3,000×, and 10,000× (HepG2), and 3,000×, 10,000×, and 20,000× (LX2 and CCS). Scale bars are shown in each panel

To further characterize the structural organization of the spheroid models, scanning electron microscopy (SEM) was performed after 96 h of culture (Fig. 4). SEM analysis was consistent with the distinct ultrastructural features previously identified by morphometric analysis. LX-2 spheroids exhibited a compact and continuous outer surface with relatively smooth contours and few surface irregularities. In contrast, HepG2 spheroids displayed a markedly heterogeneous architecture characterized by a rough, porous surface composed of loosely associated cellular aggregates. CCS spheroids exhibited an intermediate phenotype, presenting a compact external organization like LX-2 spheroids while maintaining discrete surface irregularities, suggesting the contribution of the stromal compartment to spheroid organization.

Higher magnification images further highlighted these differences. LX-2 spheroids displayed a dense and homogeneous surface, whereas HepG2 spheroids showed abundant pores and an irregular three-dimensional network. CCS spheroids exhibited a more homogeneous surface than HepG2 spheroids, although small irregularities remained detectable. Overall, the SEM observations corroborated the morphometric findings, indicating that CCS displayed structurally organized spheroids while preserving ultrastructural characteristics associated with stromal cells.

### Cell death

The marked differences in spheroid architecture observed during morphometric analysis suggested that each culture model exhibited a distinct biological behavior. Because spheroid compactness may influence cell survival, Annexin V-FITC/PI staining was performed after 96 h of spheroid formation to compare the cell-death profile among HepG2, LX-2 and CCS spheroids.

**Fig. 5:**
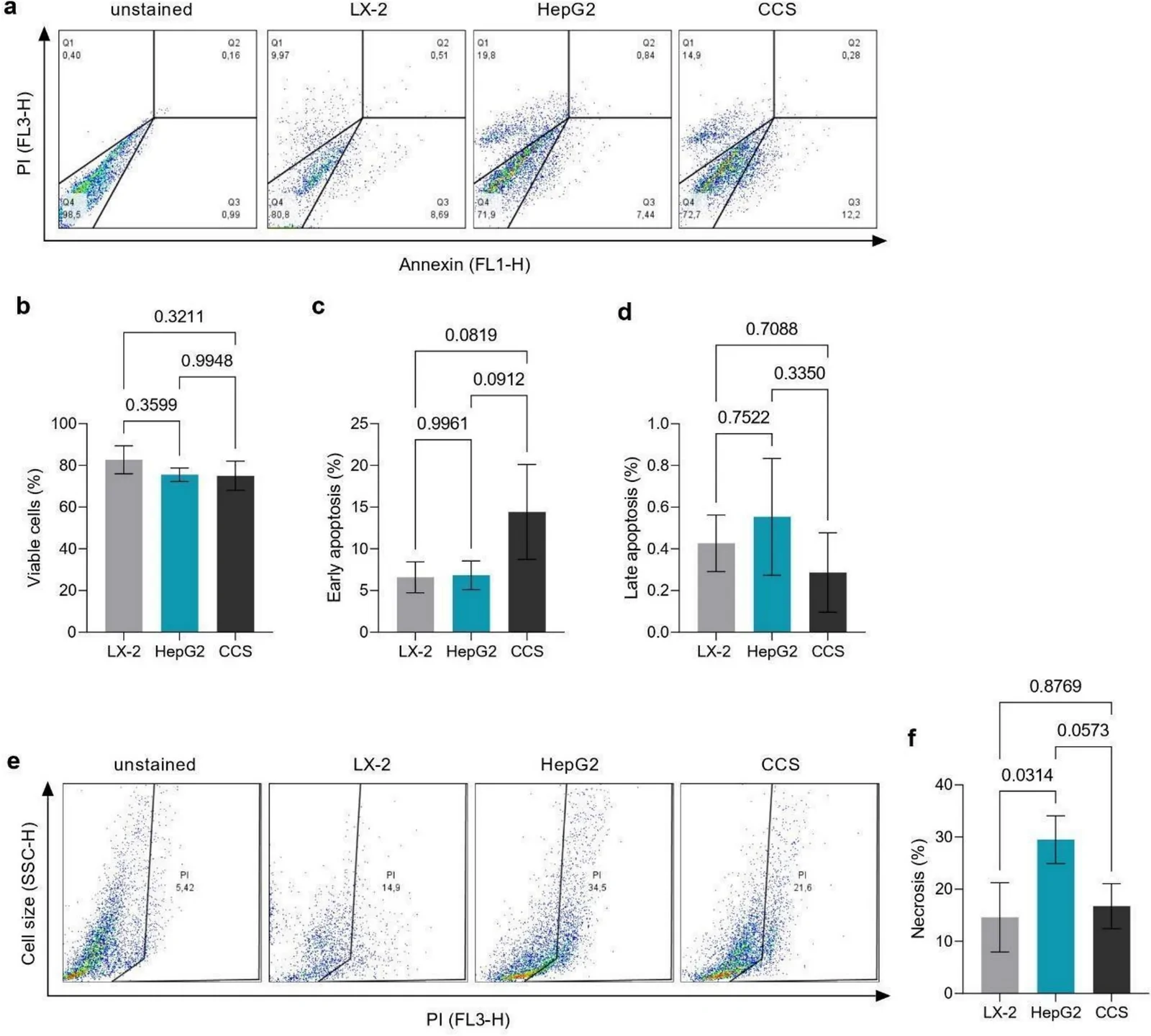
Flow cytometric characterization of cell-death profiles in LX-2, HepG2, and CCS spheroids after 96 h of culture. (**a**) Representative Annexin V-FITC/propidium iodide (PI) flow cytometry plots**.** Quadrant were defined as follows: Annexin⁻/PI⁺ (Q1), Annexin V+/PI+ late apoptotic or membrane-compromised cells (Q2), Annexin V+/PI− early apoptotic cells (Q3), and Annexin V−/PI− viable cells (Q4). Unstained cells were used for gate definition. (**b-d**) Quantification of the proportions of viable cells (**b**), early apoptotic cells (**c**), and late apoptotic cells (**d**) in the spheroids. (**e**) Representative SSC-H versus PI fluorescence dot plots showing the gate used to quantify total PI-positive cells. Total PI positivity was defined as the sum of the Annexin V−/PI+ and Annexin V+/PI+ populations (Q1 + Q2) and was used as an indicator of plasma membrane compromise. (**f**) Quantification of total PI-positive cells. Data are presented as mean ± standard deviation from three independent biological replicates (n=3), each consisting of ten pooled spheroids per experimental group. Statistical comparisons were performed using one-way ANOVA followed by Tukey’s multiple-comparisons test. Exact p values are shown in the figure

The overall cell-death profiles were broadly similar among the spheroid models after 96 h of culture. The proportion of viable cells (Annexin−/PI−; Q4) did not differ between LX-2 and HepG2 spheroids (*p* = 0.3599), LX-2 and CCS spheroids (*p* = 0.3211), or HepG2 and CCS spheroids (*p* = 0.9948; Fig. 5a,b). Early apoptotic cells (Annexin+/PI−; Q3) also showed no statistically significant differences among the spheroid models. Pairwise comparisons yielded *p* = 0.9961 for LX-2 versus HepG2, *p* = 0.0819 for LX-2 versus CCS, and p=0.0912 for HepG2 versus CCS (Fig. 5a). Although CCS spheroids exhibited the highest mean proportion of early apoptotic cells, this difference did not reach statistical significance. Similarly, the proportion of late apoptotic cells (Annexin V+/PI+; Q2) did not differ between LX-2 and HepG2 spheroids (*p* = 0.7522), LX-2 and CCS spheroids (*p* = 0.7088), or HepG2 and CCS spheroids (*p* = 0.3350; Fig. 5a, d).

In contrast, differences emerged when PI-positive cells were evaluated as an indicator of plasma membrane compromise. HepG2 spheroids exhibited a significantly higher proportion of PI-positive cells than LX-2 spheroids (*p* = 0.0314; Fig. 5e, f). The proportion was also higher in HepG2 than in CCS spheroids although this comparison did not reach statistical significance (*p* = 0.0573). No difference was detected between LX-2 and CCS spheroids (*p* = 0.8769).

**Fig. 6:**
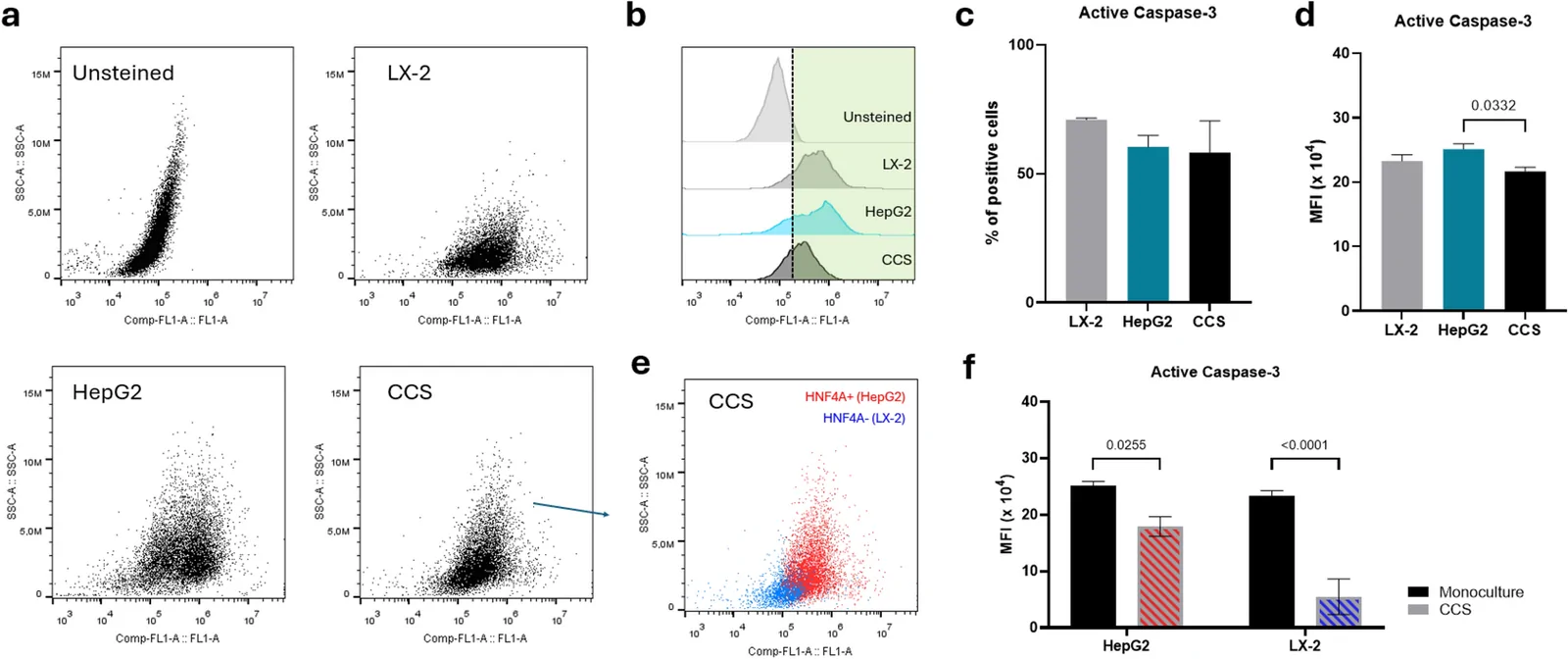
Flow cytometric assessment of active caspase−3 abundance in HepG2, LX-2, and CCS spheroids. (**a**) Representative SSC-A versus Alexa Fluor® 488 fluorescence plots showing active caspase−3 staining in unstained cells, LX-2 monocultures, HepG2 monocultures, and CCS spheroids. (**b**) Representative overlaid histograms showing the distribution of active caspase−3 fluorescence in the different spheroid models. The dashed line indicates the positivity threshold established using the matched active caspase−3 secondary-antibody-only control. (**c**) Percentage of active caspase−3-positive cells within the FSC/SSC-selected singlet population. (**d**) Active caspase−3 MFI calculated across the entire singlet population, irrespective of the positivity threshold. (**e**) Representative back-gating plot showing active caspase−3 fluorescence in HNF4α-positive and HNF4α-negative populations from CCS spheroids. Cells were initially classified according to HNF4α fluorescence and subsequently displayed on an SSC-A versus active caspase−3–Alexa Fluor 488 fluorescence plot. HNF4α-positive cells are shown in red and were operationally classified as HepG2-like cells, whereas HNF4α-negative cells are shown in blue and were classified as putative LX-2-like cells. (**f**) Active caspase−3 MFI in HepG2 and LX-2 monocultures and in the corresponding HNF4α-positive and HNF4α-negative populations identified within CCS spheroids. For monocultures, fluorescence intensity was calculated within the total singlet population; for CCS spheroids, it was calculated separately within the HNF4α-positive and HNF4α-negative singlet gates. Data are presented as mean ± SD from three independent biological replicates (n=3), each consisting of 6–8 pooled spheroids. Panels (c) and (d) were analyzed using one-way ANOVA followed by Tukey’s multiple-comparisons test. Panel (f) was analyzed using two-way ANOVA followed by Šídák’s multiple-comparisons test, with comparisons performed between each monoculture and its corresponding CCS-derived population. Exact p values are shown in the figure

Active caspase−3 immunofluorescence was subsequently assessed to complement the phenotypic cell-death profile obtained by Annexin V/PI staining. All analyses were performed within the singlet-cell population after sequential exclusion of debris and doublets, as shown in Supplementary Fig. S2. Representative histograms showed active caspase−3 staining in all spheroid models relative to the matched negative control (Fig. 6a,b). The percentage of active caspase−3-positive singlet cells did not differ significantly among LX-2, HepG2, and CCS spheroids (Fig. 6c). However, when active caspase−3 abundance was quantified as MFI across the entire singlet population, CCS spheroids exhibited a lower signal than HepG2 spheroids (*p* = 0.0332), whereas no other significant differences were detected among the spheroid models (Fig. 6d).

To investigate whether the coculture environment differentially affected the two cell-associated populations, singlet cells from CCS spheroids were first classified according to HNF4α immunofluorescence. HNF4α-positive cells were operationally identified as HepG2-like cells, whereas HNF4α-negative cells were classified as the putative LX-2-like population (Supplementary Fig. S5). These HNF4α-defined populations were subsequently back-gated onto the active caspase−3 fluorescence plot, allowing active caspase−3 MFI to be quantified separately within each population (Fig. 6e). Compared with the corresponding monocultures, the HNF4α-positive HepG2-like population within CCS spheroids exhibited an approximately 28% lower median active caspase−3 fluorescence intensity than HepG2 monocultures (p = 0.0255). Similarly, the putative LX-2-like HNF4α-negative population within CCS spheroids showed an approximately 76% lower signal than LX-2 monocultures (*p* < 0.0001; Fig. 6f). Percentage reductions were calculated from the corresponding group median fluorescence intensities.

### Membrane integrity and extracellular metabolic profile

**Fig. 7:**
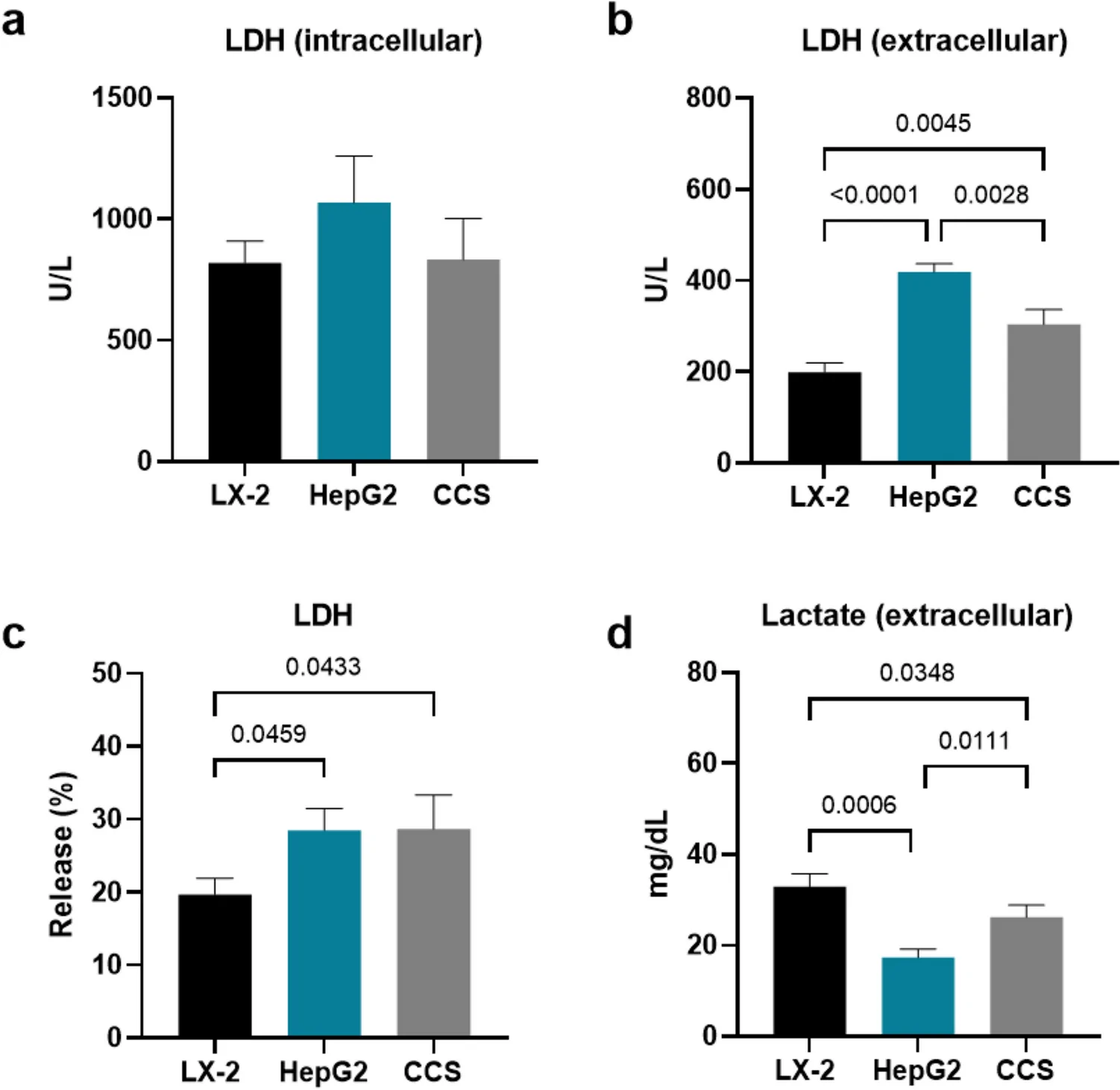
Biochemical assessment of membrane integrity and extracellular lactate concentrations in LX-2, HepG2 and CCS spheroids. (**a**) Intracellular and (**b**) extracellular lactate dehydrogenase (LDH) activity expressed as U/L. (**c**) Percentage of LDH release, calculated as extracellular LDH relative to total LDH activity. (**d**) Extracellular lactate concentration expressed as mg/dL. Data are presented as mean ± SD from three independent biological replicates (n = 3), each consisting of four pooled spheroids. Statistical significance was determined by one-way ANOVA followed by Tukey’s multiple-comparisons test. Exact p values are indicated in the figure

Biochemical analyses revealed distinct extracellular LDH and lactate profiles among the spheroid models after 96 h of culture. Intracellular and extracellular LDH activities, percentage LDH release, and extracellular lactate concentration were evaluated, and the kinetic measurements used to calculate LDH activity are shown in Supplementary Fig. S3. Intracellular LDH activity did not differ significantly among HepG2, LX-2, and CCS spheroids (Fig. 7a). In contrast, extracellular LDH activity differed among the spheroid models (Fig. 7b). HepG2 spheroids exhibited higher extracellular LDH activity than LX-2 (*p*<0.0001) and CCS spheroids (*p* = 0.0028). CCS spheroids also exhibited higher extracellular LDH activity than LX-2 spheroids (*p* = 0.0045). The percentage of LDH release was higher in HepG2 and CCS spheroids than in LX-2 spheroids (*p* = 0.0459 and *p* = 0.0433, respectively), whereas no significant difference was detected between HepG2 and CCS spheroids (Fig. 7c). Thus, although extracellular LDH activity was lower in CCS than in HepG2 spheroids, the calculated proportion of LDH released did not differ between these two models. Extracellular lactate concentration also differed among the spheroid models (Fig. 7d). LX-2 spheroids exhibited a higher lactate concentration in conditioned medium than HepG2 (*p* = 0.0006) and CCS spheroids (*p* = 0.0348). CCS spheroids also exhibited a higher extracellular lactate concentration than HepG2 spheroids (*p*=0.0111).

### α-SMA-defined cellular fractions and COL1A1 abundance

**Fig. 8:**
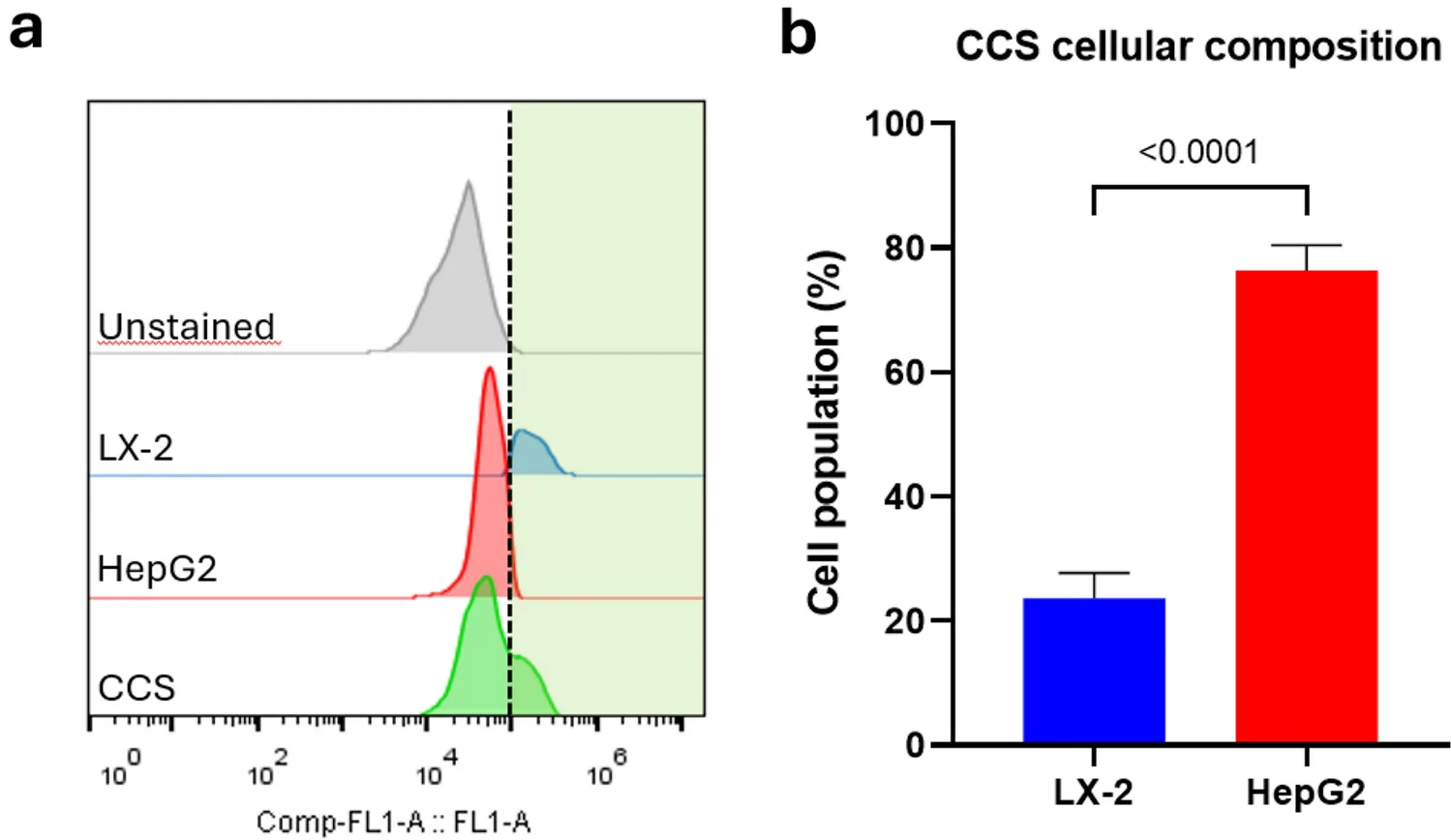
α-SMA-defined cellular fractions within coculture system (CCS) spheroids. (**a**) Representative overlaid histograms showing α-SMA–Alexa Fluor® 488 fluorescence in unstained cells, LX-2 monocultures, HepG2 monocultures, and CCS spheroids. The dashed line indicates the threshold used to distinguish α-SMA-positive and α-SMA-negative cells, established relative to the unstained control. (**b**) Proportions of α-SMA-positive and α-SMA-negative singlet cells within CCS spheroids after 96 h of culture. The α-SMA-positive fraction was interpreted as LX-2-like and stromal-associated, whereas the α-SMA-negative fraction was considered predominantly HepG2-like. Data are presented as mean ± SD from three independent biological replicates (n = 3), each consisting of 6–8 pooled spheroids

α-SMA immunofluorescence revealed two marker-defined fractions within CCS spheroids. LX-2 monocultures showed a greater proportion of α-SMA-positive cells than HepG2 monocultures, supporting the use of α-SMA as a stromal-associated marker in this coculture model (Fig. 8a). Within CCS spheroids, approximately 24% of singlet cells were classified as α-SMA-positive, whereas approximately 76% were α-SMA-negative after 96 h of culture (Fig. 8b). The α-SMA-positive fraction was interpreted as an LX-2-like, stromal-associated population, while the α-SMA-negative fraction was considered predominantly HepG2-like. However, because α-SMA reflects marker expression and activation state rather than definitive lineage, these percentages represent α-SMA-defined fractions and not an exact measurement of the cellular composition of CCS spheroids.

**Fig. 9:**
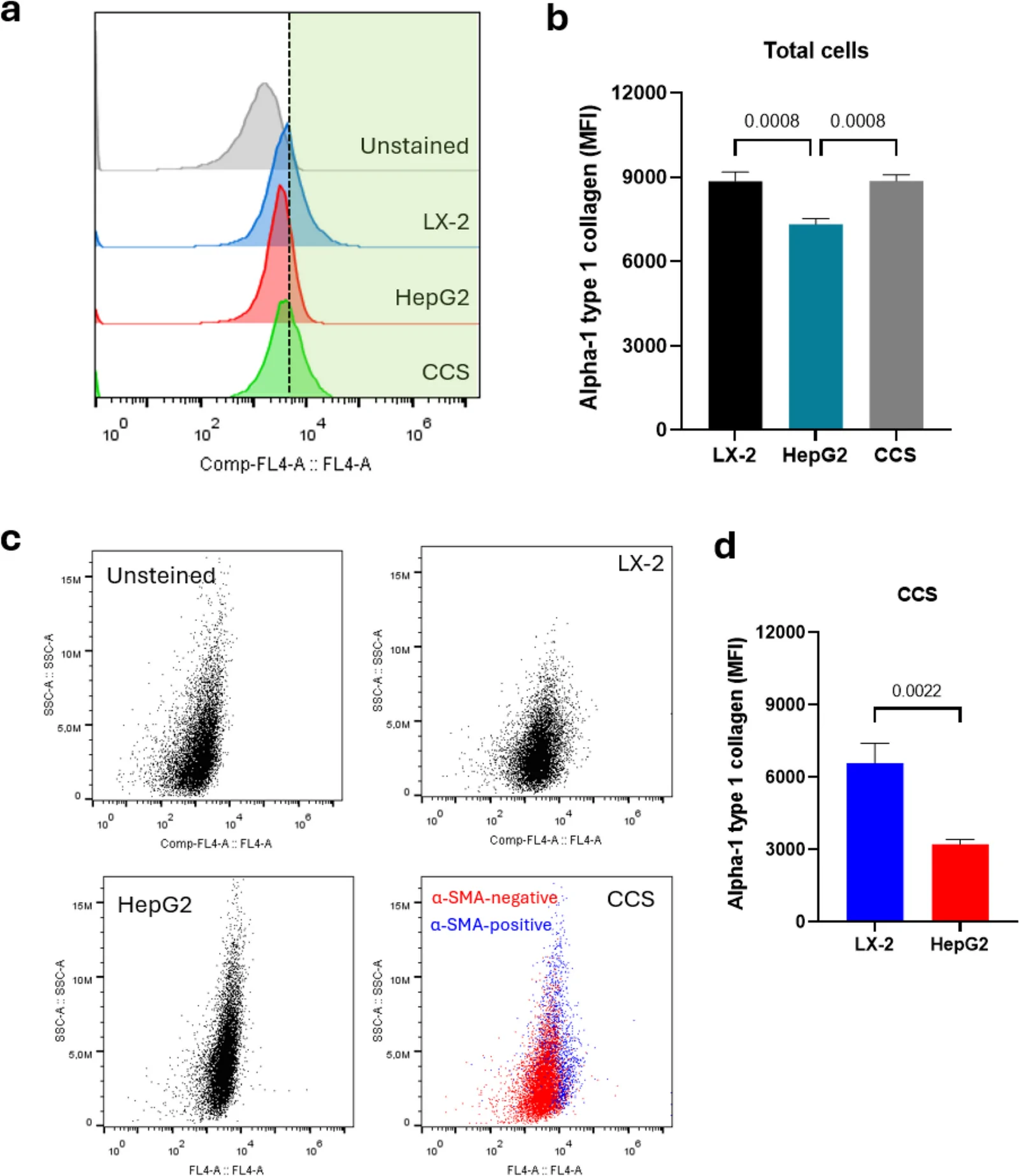
Flow cytometric analysis of COL1A1 expression in HepG2, LX-2, and CCS spheroids. (**a**) Representative flow cytometry fluorescence distributions showing COL1A1-associated staining in HepG2, LX-2, and CCS spheroids. (**b**) Corrected MFI of COL1A1 calculated across the entire singlet-cell population of each spheroid model. (**c**) Representative gating of α-SMA-positive and α-SMA-negative fractions within the CCS singlet-cell population. In the context of the HepG2/LX-2 coculture, the α-SMA-positive fraction was interpreted as stromal-associated and LX-2-like, whereas the α-SMA-negative fraction was considered predominantly HepG2-like. (**d**) Corrected MFI of COL1A1 was calculated separately within the α-SMA-positive and α-SMA-negative fractions of CCS spheroids. Corrected MFI was calculated by subtracting the MFI of the matched secondary-antibody-only control from that of the stained sample. Data are presented as mean ± SD from three independent biological replicates (n=3), each consisting of 6–8 pooled spheroids. Panel (b) was analyzed using one-way ANOVA followed by Tukey’s multiple-comparisons test. Panel (d) was analyzed using a paired two-tailed t-test. Exact p values are shown in the figure

To further characterize the matrix-associated phenotype of the spheroid models, COL1A1 immunofluorescence was quantified by flow cytometry. Corrected MFI of COL1A1, calculated across the entire singlet-cell population, differed significantly among the spheroid models (Fig. 9a,b). HepG2 spheroids exhibited lower COL1A1 MFI than both LX-2 and CCS spheroids (*p* = 0.0008 for both comparisons). No significant difference was detected between LX-2 and CCS spheroids, indicating that the overall COL1A1-associated signal in CCS was comparable to that observed in LX-2 monocultures. The distribution of this signal within CCS spheroids was then examined by stratifying the singlet population into α-SMA-positive and α-SMA-negative fractions (Fig. 9c). In the context of the HepG2/LX-2 coculture, the α-SMA-positive fraction was interpreted as stromal-associated and LX-2-like, whereas the α-SMA-negative fraction was considered predominantly HepG2-like. Corrected MFI of COL1A1 was significantly higher in the α-SMA-positive fraction than in the α-SMA-negative fraction (*p* = 0.0022; Fig. 9d). These findings indicated that the COL1A1-associated signal within CCS spheroids was predominantly associated with the α-SMA-positive, stromal-like fraction.

### Coculture spheroids exhibit reduced sensitivity to sorafenib

**Fig. 10:**
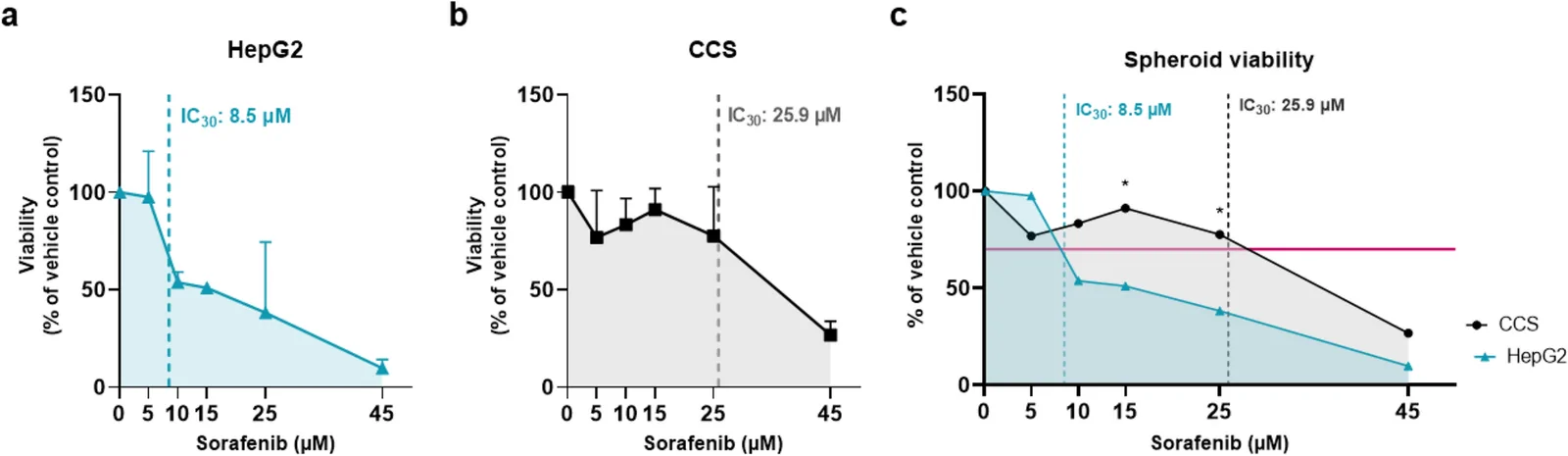
Effect of sorafenib on the viability of HepG2 and CCS spheroids. HepG2 (**a**) and CCS (**b**) spheroids were exposed to increasing concentrations of sorafenib (0–45 μM) for 48 h, and viability was assessed using the CellTiter-Glo® 3D assay. Vehicle-control values were set to 100%. The superimposed concentration–response profiles are shown in panel c. The horizontal line indicates 70% viability, corresponding to the threshold used to determine the IC₃₀, while the dashed vertical lines indicate the estimated IC₃₀ values for HepG2 (8.5 μM) and CCS (25.9 μM) spheroids. Data in panels a and b are presented as mean ± SD from three independent experiments; panel c shows the corresponding mean values. Statistical differences were evaluated by two-way ANOVA followed by Šídák’s multiple-comparisons test. Significant effects of sorafenib concentration (p < 0.0001), spheroid model (p = 0.0050), and their interaction (p = 0.0306) were observed. CCS viability was significantly higher than HepG2 viability at 15 and 25 μM sorafenib (adjusted *p* = 0.0478, and *p* = 0.05, respectively)

The structural and biochemical differences observed between HepG2 and CCS spheroids suggested that the stromal compartment could influence therapeutic response. To investigate this possibility, spheroid viability was evaluated after exposure to increasing concentrations of sorafenib for 48 h using the CellTiter-Glo® 3D assay. HepG2 spheroids maintained viability comparable to the vehicle control at 5 μM, with a marked reduction observed from 10 μM onward. (Fig. 10a). In contrast, CCS spheroids maintained mean viability above 70% up to 25 μM, with a pronounced reduction observed only at the highest concentration tested (Fig. 10b). Two-way ANOVA revealed a significant interaction between sorafenib concentration and spheroid model (*p* = 0.0306), demonstrating that the response to sorafenib depended on spheroid composition. Significant main effects of sorafenib concentration (*p* < 0.0001) and spheroid model (*p* = 0.0050) were also observed. Šídák’s multiple-comparisons test showed significantly higher viability in CCS than in HepG2 spheroids at 15 and 25 μM sorafenib (*p* = 0.0478 and *p* = 0.05, respectively), whereas no significant differences were detected at the remaining concentrations (Fig. 10c). Concentration–response analysis yielded an estimated IC₃₀ of 8.5 μM for HepG2 spheroids and 25.9 μM for CCS spheroids. Thus, the estimated IC₃₀ was approximately threefold higher in CCS spheroids, supporting reduced sensitivity to sorafenib in the presence of LX-2 cells.

## Discussion

While in vitro HCC models have advanced our understanding of tumor biology and therapeutic responses, their predictive value is often limited using traditional 2D monocultures, which lack the complexity of the in vivo TME [35]. In contrast, 3D culture systems (particularly cocultures) offer more physiologically relevant tumor models, more accurately mimicking the TME [36], including cell–cell interactions and ECM production [37]. 3D coculture models have consistently demonstrated that HSCs actively regulate HCC spheroid organization through ECM remodeling, stromal activation, and paracrine signaling [19, 38, 39]. However, despite increasing evidence that stromal cells influence spheroid architecture, relatively few studies have comprehensively characterized how these structural changes are accompanied by alterations in ECM production, cell death, metabolic profile, and therapeutic response. In the present study, we combined morphometric, ultrastructural, molecular, biochemical, and functional analyses to provide an integrated characterization of HepG2/LX-2 spheroids. Overall, the incorporation of LX-2 cells was associated with a more compact morphometric profile, altered active caspase−3 and extracellular LDH/lactate signals, increased COL1A1-associated fluorescence, and reduced sorafenib sensitivity. CCS spheroids also showed a lower mean PI-positive fraction than HepG2 spheroids, although this comparison did not reach statistical significance. These observations identify phenotypic differences among the spheroid models but do not establish the specific structural, metabolic, or signaling mechanisms responsible.

Morphometric parameters such as area, perimeter, circularity, and roundness are commonly used to evaluate the size, shape, and regularity of biological structures, including adipocytes [40] and cell nuclei [41]. These measurements have also proven helpful in assessing spheroid formation across different 3D culture methodologies [18, 42], as they offer quantitative insights into the structural organization of these models. Consistent with this approach, our morphometric analysis revealed distinct growth and organizational patterns among HepG2, LX-2, and CCS spheroids, indicating that the incorporation of HSCs substantially influences spheroid architecture during its earliest stages of assembly.

Our morphometric analysis encompassed multiple shape descriptors, including area, perimeter, circularity, roundness, aspect ratio, solidity (geometric and normalized), and density. While roundness, aspect ratio, and geometric solidity did not differ significantly across groups, circularity showed a distinct pattern: HepG2 spheroids exhibited significantly lower circularity than LX-2 and CCS spheroids. Although circularity and roundness are related, they describe different geometric attributes, with circularity being more sensitive to contour irregularities and symmetry, whereas roundness predominantly reflects elongation [23]. The reduced circularity observed in HepG2 spheroids suggests greater structural irregularity or asymmetry, which could influence internal diffusion dynamics.

It has been shown that spheroid geometry directly affects diffusion gradients, since circular and symmetric spheroids facilitate more uniform diffusion toward the center, whereas irregular or elongated shapes increase surface area and reduce the average diffusion distance, potentially leading to heterogeneous oxygen, nutrients, and drug distribution [43, 44]. The lower circularity of HepG2 spheroids may contribute to a more heterogeneous spatial distribution of oxygen, nutrients, and metabolites. However, their larger size is likely to be more directly relevant to the development of diffusion limitations and the greater proportion of membrane-compromised cells observed in this model. Although circularity alone does not define compactness, it reflects geometric properties that are functionally relevant in spheroid biology [45].

Distinct trends were also observed in size-related parameters. Both area and perimeter progressively decreased in LX-2 and CCS spheroids throughout the culture period, suggesting rapid and sustained compaction, while HepG2 spheroids exhibited an increase in diameter during the first 24 h (Supplementary Fig. S1), resulting in a larger overall structure. Although spheroid area is frequently interpreted as an indicator of growth [46], our findings suggest that it should also be interpreted in the context of structural organization, since reductions in area may primarily reflect increased compactness rather than reduced proliferation. This distinction is particularly important during the early stages of spheroid maturation, when extensive cellular rearrangement occurs.

Compactness in LX-2 and CCS spheroids was further supported by increased normalized solidity, reduced MGV, and reduced area and perimeter. These quantitative observations were corroborated by scanning electron microscopy, which revealed distinct ultrastructural organization among the spheroid models. HepG2 spheroids exhibited a rough and heterogeneous external architecture, on the other hand CCS spheroids displayed a more continuous and compact surface organization, consistent with the enhanced structural cohesion indicated by the morphometric analysis. The presence of LX-2 cells therefore appears to enhance mechanical stability and spheroid compaction, most likely through ECM production and stromal-tumor interactions ^20^. Our results are consistent with previous studies showing that stromal cells promote spheroid cohesion and maturation, supporting the concept that CCS spheroid morphology results from active tumor-stromal interactions rather than passive cell aggregation [47, 48].

Our observations are broadly consistent with previous reports describing the structural evolution of HCC spheroids, although methodological differences should be considered. Taroncher et al. [49] observed continued spheroid growth in cocultures during the final 24 h of a 96-h culture period, whereas our normalized area analysis revealed no significant growth after 72 h (Supplementary Fig. S4). This discrepancy could be attributable to differences in spheroid formation protocols, as their model employed centrifugation to accelerate cell aggregation, while spheroids in the present study formed through spontaneous self-assembly. Although spontaneous aggregation results in slower spheroid formation, it more closely reflects the natural dynamics of cellular organization and represents a technically accessible approach for generating reproducible multicellular spheroids.

Likewise, geometric solidity values obtained for HepG2 spheroids (80.7%) were comparable to those reported in the literature (80–95%) [49], supporting the robustness of our morphometric analysis. Interestingly, normalized solidity and MGV proved more informative than geometric solidity for distinguishing stromal-induced differences in spheroid organization, revealing significant differences between HepG2 and stromal-containing spheroids. Because these normalized descriptors account for inter-spheroid variability, they may provide greater sensitivity for detecting subtle changes in spheroid compaction and structural maturation. These findings further support the use of quantitative morphometric parameters as sensitive tools for evaluating stromal-mediated architectural remodeling in 3D HCC models.

Consistent with these structural differences, CCS spheroids also exhibited fewer membrane-compromised cells than HepG2 monocultures, indicating that the stromal-induced architectural remodeling was accompanied by improved spheroid integrity. A study using a live/dead fluorescence assay showed similar results, with coculture spheroids demonstrating significantly higher viability than HepG2 monocultures [49]. The authors attributed this to stellate cells improving hepatocyte viability and structural organization. Together, our findings demonstrate that spheroid morphology and viability are strongly influenced by cellular composition. Coculture with LX-2 cells promoted more compact, symmetrical, and viable spheroids, better representing the TME. The structural remodeling promoted by HSCs was accompanied by significant differences in membrane-compromised cells. Among the cell death parameters evaluated, PI-positive cells were the only population that differed significantly among the spheroid models, with HepG2 monoculture spheroids exhibiting the highest proportion of membrane-compromised cells. This finding is consistent with the distinct structural organization observed throughout spheroid formation. Larger spheroids are more susceptible to diffusion limitations, and those exceeding approximately 500 μm in diameter develop pronounced oxygen and nutrient gradients that promote central hypoxia and extensive cell death [24]. Although all spheroid models in our study exceeded this size threshold, their structural evolution differed substantially. CCS and LX-2 spheroids underwent progressive compaction, and HepG2 spheroids maintained a larger and less cohesive architecture. The reduced proportion of PI-positive cells in CCS spheroids may therefore reflect survival signals generated by tumor–stromal interactions and ECM-associated signaling rather than spheroid compaction alone. These findings align with previous studies demonstrating that HSCs support hepatocyte viability and spheroid integrity via the secretion of ECM and survival factors [50, 51]. By enhancing compactness and possibly contributing ECM components, LX-2 cells modulate the physical and biochemical environment, supporting cell survival [52]. This highlights the value of CCS in recapitulating the tumor-stromal interplay seen in vivo [53]. The reduced proportion of membrane-compromised cells observed in CCS spheroids reflects not only the presence of additional stromal cells but also the establishment of a more cohesive and supportive microenvironment capable of preserving spheroid integrity.

The use of LX-2 cells should be interpreted in the context of their constitutively activated phenotype. Although quiescent HSCs predominate in healthy liver, chronic liver injury and fibrosis promote their trans differentiation into myofibroblast-like cells characterized by increased ECM production, particularly COL1 [54]. This activated stromal phenotype is highly relevant to HCC, as most tumors arise in chronically injured, fibrotic, or cirrhotic livers, where activated HSCs are major regulators of the TME 2. In our model, the greater proportion of α-SMA-positive cells in LX-2 monocultures and the enrichment of COL1A1-associated fluorescence within the α-SMA-positive compartment of CCS spheroids support the presence of an activated-like, collagen-producing stromal phenotype. Therefore, the morphometric, cell-death-associated, and pharmacological characteristics of CCS spheroids should be interpreted as responses occurring in the presence of activated-like HSCs rather than HSCs in general. To investigate a potential matrix-related component of the structural organization observed in CCS spheroids, we quantified intracellular COL1A1-associated fluorescence by flow cytometry. Flow-cytometric analysis demonstrated that COL1A1 expression was significantly higher in CCS than in HepG2 spheroids and that COL1A1 expression was predominantly detected within the α-SMA-positive population, identifying the α-SMA-positive population as the predominant source of COL1A1 in the CCS model. These findings provide experimental evidence that stromal cells actively contribute to ECM production within CCS spheroids suggesting that HSC-derived ECM may contribute to spheroid compaction [19, 38]. Previous studies have demonstrated that HSCs actively remodel the ECM through the production of COL1A1 and other matrix components, promoting spheroid cohesion, increasing tissue stiffness, and altering tumor architecture [3, 55, 56]. In multicellular HCC spheroids, HSC-derived COL1A1 has been shown to increase compactness and chemoresistance [38]. Also, inhibition of collagen deposition reduces spheroid cohesion and improves therapeutic penetration [19]. These findings support the possibility that HSC-derived ECM contributes to the increased structural cohesion of CCS spheroids, although direct measurements of collagen deposition and matrix mechanics would be required to establish this relationship.

Spheroid composition was also associated with differences in active caspase−3 MFI. Although the percentage of active caspase−3-positive cells was similar among the spheroid models, active caspase−3 fluorescence intensity, measured as corrected MFI, was higher in HepG2 monoculture spheroids than in the corresponding HNF4α-positive population within CCS spheroids. Thus, coculture did not significantly alter the frequency of cells displaying detectable caspase−3 activation but reduced the magnitude of the active caspase−3 signal within the analyzed cell population. Separate analysis of the cellular compartments further supported this interpretation, as both HNF4α-positive and HNF4α-negative populations exhibited lower corrected MFI in CCS spheroids than in their respective monocultures. This reduction was particularly pronounced in the HNF4α-negative, putative LX-2-like compartment, suggesting that stellate-like cells may be especially responsive to survival signals generated within the multicellular spheroid microenvironment. The susceptibility of hepatic stellate cells to apoptosis is highly context dependent and can be modulated by extracellular matrix composition, integrin-mediated signaling, and paracrine interactions that regulate the balance between cell survival and programmed cell death [54]. Accordingly, the reduced active caspase−3 fluorescence intensity observed in CCS spheroids is consistent with attenuation of caspase−3-mediated apoptotic execution rather than complete prevention of caspase−3 activation. This interpretation agrees with studies showing that stromal cells in 3D CCS can limit cell-death responses through heterotypic cell–cell interactions, ECM remodeling, and activation of pro-survival signaling pathways [57, 58]. Nevertheless, because caspase−3 activation is transient and time dependent, reduced fluorescence intensity alone cannot definitively establish reduced apoptosis. Moreover, because the putative LX-2-like compartment was identified by the absence of HNF4α staining, the inclusion of additional cell-type-specific markers would strengthen the assignment of the HNF4α-negative population to LX-2 cells.

The differences in cell membrane integrity identified by flow cytometry were accompanied by distinct patterns of extracellular LDH activity, although the normalized LDH release results did not completely reproduce the differences observed in the PI-positive populations. Since LDH is released following disruption of plasma membrane integrity, extracellular LDH activity is widely used as an indicator of cell membrane damage and cytotoxicity [59]. Biochemical analyses revealed distinct patterns of LDH distribution among the spheroid models. Intracellular LDH activity did not differ significantly, whereas extracellular LDH activity was highest in HepG2 spheroids, intermediate in CCS spheroids, and lowest in LX-2 spheroids. The higher extracellular LDH activity in HepG2 than in CCS was consistent with the greater proportion of PI-positive cells observed in the HepG2 monoculture. However, when LDH release was expressed relative to the total measurable LDH activity, both HepG2 and CCS spheroids exhibited higher release percentages than LX-2 spheroids, with no significant difference between HepG2 and CCS. Thus, although the extracellular LDH values partially agree with the flow-cytometry results, the normalized LDH release does not provide direct evidence of greater membrane preservation in CCS relative to HepG2. This apparent discrepancy may reflect the distinct temporal characteristics of the assays, as PI staining identifies cells with compromised plasma membranes at the time of analysis, whereas extracellular LDH activity reflects LDH released into the culture medium before sample collection [60, 61].

Extracellular lactate also differed significantly among all spheroid models, with LX-2 spheroids displaying the highest concentration, HepG2 spheroids the lowest, and CCS spheroids an intermediate level. This pattern indicates that the incorporation of LX-2 cells shifts the extracellular metabolic profile of the coculture away from that of HepG2 monocultures without completely reproducing the phenotype of LX-2 spheroids. This interpretation is biologically plausible because hepatic stellate-cell activation can involve LDH-A- and HIF-1α-dependent glycolytic reprogramming and increased lactate production [62]. However, extracellular lactate represents the net balance among intracellular production, membrane export, cellular uptake, and subsequent utilization rather than a direct measurement of glycolytic flux. In HepG2 cells, both MCT1 and MCT4 contribute to lactate transport, demonstrating that extracellular lactate levels may also be influenced by lactate uptake and exchange across the plasma membrane [63]. Furthermore, experimental studies have shown that stromal cells can either export lactate or reutilize tumor-derived lactate, supporting the existence of bidirectional metabolic interactions between tumor and stromal compartments [64, 65]. Therefore, the intermediate lactate concentration observed in spheroids suggests that coculture establishes a distinct metabolic state arising from the combined and potentially interactive behavior of HepG2 and LX-2 cells. Because total cell numbers did not differ significantly among the spheroid models, these differences cannot be explained simply by variations in cell abundance. Nevertheless, measurements of glucose consumption, intracellular lactate, and MCT1/MCT4 expression or activity would be required to determine the mechanisms underlying the extracellular lactate profiles.

Taken together, the structural, biochemical, metabolic, and functional findings presented here highlight the broader utility of quantitative morphometric analysis. Rather than representing purely geometric descriptors, morphometric parameters such as circularity, compactness, solidity, and density capture biologically meaningful changes associated with stromal remodeling. Several studies have shown that alterations in spheroid architecture are accompanied by ECM remodeling, epithelial-mesenchymal transition (EMT), migration, invasion, and therapeutic response [66, 67]. Accordingly, quantitative morphometric descriptors may provide valuable information regarding the biological state of HCC spheroids beyond simple geometric characterization because they reflect changes in cell organization and tissue remodeling [68, 69].

While the present study was not designed to investigate invasion, migration, or ECM dynamics directly, the distinct morphometric profiles observed between HepG2 monocultures and CCS spheroids suggest that cellular composition is a major determinant of spheroid organization and influence in tumor-associated phenotypes. Morphometric change alone does not prove invasion, because larger areas can also reflect loss of integrity or reduced compactness rather than proliferative or invasive gain [70]. The main point of quantitative morphometric analysis lies in its use as an initial screening approach that should be complemented by ECM, EMT, migration, or drug-penetration assays to establish the underlying biological mechanisms [70, 71]. Overall, our findings, together with previous reports, supports the concept that circularity, compactness, solidity, and density can serve as indirect readouts of tumor-stroma interactions in HCC spheroids.

The functional relevance of the structural and biochemical differences between the spheroid models was further demonstrated by their distinct responses to sorafenib. CCS spheroids exhibited significantly higher viability than HepG2 spheroids at 15 and 25 μM, while the significant interaction between spheroid model and sorafenib concentration indicated that the treatment response depended on spheroid composition. Consistent with these findings, the estimated IC₃₀ was approximately threefold higher in CCS than in HepG2 spheroids (25.9 versus 8.5 μM). The detection of differences at two intermediate concentrations strengthens the evidence that the presence of LX-2 cells reduces spheroid responsiveness to sorafenib. Similar findings were reported by Song et al. [19], who showed that the inclusion of LX-2 cells increased compactness and reduced sorafenib-induced cell death in HepG2 multicellular tumor spheroids. Khawar et al. [38] also observed reduced drug sensitivity in stroma-rich Huh-7/LX-2 spheroids characterized by increased COL1 and profibrotic signaling. More recently, pharmacological reprogramming of activated HSCs reduced ECM-related protein expression and improved sorafenib efficacy in multicellular HCC spheroids [39]. Nevertheless, the absence of a significant difference at the highest concentration in the present study suggests that the protective effect conferred by the stromal compartment was relative and concentration-dependent rather than indicative of complete resistance.

The reduced sensitivity of CCS spheroids may be associated with their more compact architecture and stromal remodeling. Increased COL1A1 content and the predominant detection of it in α-SMA-positive cells support the contribution of LX-2 cells to the establishment of an ECM-rich microenvironment. In a comparable HepG2/LX-2 model, COL1A1 depletion reduced spheroid compactness and increased sorafenib efficacy, providing experimental evidence linking HSC-derived COL1, structural organization, and drug response [19]. Changes in HepG2 spheroid microstructure have also been shown to affect sorafenib diffusion and uptake, supporting the possibility that differences in spheroid organization influence drug accessibility [28]. However, stromal protection may not depend exclusively on physical restriction of drug distribution. LX-2 cells have been reported to promote sorafenib resistance through HGF/c-Met/Akt and JAK2/STAT3 signaling [72], while HSC-derived laminin-332 can preserve FAK-associated survival signaling and reduce the effects of sorafenib in HCC cells [73]. These studies indicate that both structural and paracrine mechanisms may contribute to stroma-mediated drug protection. The lower baseline proportion of PI-positive cells reduced extracellular LDH activity, and lower active caspase−3 signal observed in CCS spheroids are also consistent with a microenvironment that favors cell survival. However, collagen deposition and organization, matrix stiffness, intracellular sorafenib accumulation, drug penetration, and the signaling pathways described above were not directly evaluated in the present study. Therefore, although stromal remodeling was associated with reduced sorafenib sensitivity, the specific mechanisms responsible for this effect cannot be conclusively determined.

Although COL1A1 expression provided evidence of ECM production by HSCs [74] the present study did not directly quantify collagen deposition, ECM organization, or tissue stiffness. Techniques such as second harmonic generation microscopy, immunohistochemistry, or atomic force microscopy could further characterize matrix architecture and biomechanics. Likewise, although apoptosis (active caspase−3) and membrane integrity (LDH) assays complemented the Annexin V/PI analysis, other mechanisms involved in tumor-stroma interactions, including migration, invasion, and monocarboxylate transporter (MCT1/MCT4) activity, remain to be investigated. Migration and invasion are especially relevant in HCC spheroids with stellate cells because activated HSCs increased HCC migration and MMP9 in multicellular spheroids^19^. MCT1/MCT4 transporters mediate lactate/proton export, support tumor-stromal metabolic crosstalk, and have been linked to migration, invasion, multidrug resistance, and apoptosis avoidance [75].

An important limitation of this study is the exclusive use of established cell lines. HepG2 and LX-2 provide reproducibility and facilitate comparison with previous HCC coculture studies, but they do not capture the genetic, phenotypic, and interpatient heterogeneity of primary tumor and stromal populations. Therefore, the present findings should be interpreted as characteristics of this defined in vitro system. Future studies using primary HSCs, patient-derived HCC organoids, or matched tumor–stroma cultures will be required to evaluate the generalizability and translational relevance of the model. An additional limitation is the use of LX-2 cells, an immortalized HSC line with an activated-like phenotype. Although this phenotype is relevant to the fibrotic HCC microenvironment, the model does not reproduce the transition from quiescent to activated HSCs or the functional heterogeneity of stromal populations found in human tumors. Because quiescent, cytokine-producing, and myofibroblastic HSC populations may have distinct effects on hepatocyte survival and tumor progression, the present findings cannot be generalized to all HSC states. Comparisons involving primary quiescent HSCs, experimentally activated HSCs, and patient-derived tumor-associated HSCs will be necessary to determine how activation state specifically affects spheroid organization, cell-death patterns, and drug response. Despite these limitations, our approach provides a valuable foundation for future studies aiming to refine in vitro HCC models and probe tumor-stroma interactions.

In summary, our findings demonstrate that HSCs influence not only the structural organization of HCC spheroids but also their biological behavior. By promoting ECM production, increasing spheroid compactness, modulating cell death patterns, and reducing sensitivity to sorafenib, stromal cells generate a microenvironment that more closely resembles the complexity of human HCC.

## Conclusion

Longitudinal morphometric analysis provided an objective and non-invasive characterization of spheroid assembly, revealing model-specific differences in size, circularity, solidity, optical density, and compaction over 96 h. For several parameters, CCS spheroids exhibited a morphometric profile closer to that of LX-2 spheroids than to HepG2 monocultures, forming smaller, more circular, and more compact structures. These findings demonstrate that morphometric monitoring can detect dynamic changes in spheroid organization that may not be adequately represented by endpoint measurements alone, supporting its use for model characterization and quality control in 3D culture studies. The morphometric differences were accompanied by increased COL1A1 content, reduced membrane damage, and lower active caspase−3 signal in CCS spheroids, indicating that the presence of LX-2 cells influenced both structural organization and basal cell survival. Functionally, CCS spheroids exhibited significantly higher viability at 15 and 25 μM sorafenib and an estimated IC₃₀ approximately threefold higher than that of HepG2 spheroids. Although the mechanisms responsible for this reduced sensitivity remain to be determined, the combined morphometric, biochemical, and pharmacological findings demonstrate that HSCs substantially reshape the phenotype and therapeutic response of 3D HCC spheroids. Overall, these results reinforce the value of multiparametric morphometric analysis for selecting and standardizing 3D models and highlight the importance of incorporating stromal components into platforms intended to investigate TME interactions and drug response.

## Supplementary Information

Below is the link to the electronic supplementary material.

Supplementary file1 (JPG 460 KB)

Supplementary file2 (JPG 1146 KB)

Supplementary file3 (JPG 1188 KB)

Supplementary file4 (JPG 1006 KB)

Supplementary file5 (JPG 1382 KB)

Supplementary file6 (JPG 1096 KB)

Supplementary file7 (JPG 1237 KB)

Supplementary file8 (JPG 1092 KB)

Supplementary file9 (JPG 884 KB)

Supplementary file10 (JPG 803 KB)

Supplementary file11 (JPG 1784 KB)

Supplementary file12 (JPG 906 KB)

Supplementary file13 (JPG 862 KB)

Supplementary file14 (JPG 1404 KB)

Supplementary file15 (JPG 947 KB)

Supplementary file16 (JPG 1524 KB)

Supplementary file17 (JPG 3058 KB)

Supplementary file18 (JPG 164 KB)

Supplementary file19 (JPG 248 KB)

Supplementary file20 (DOCX 4666 KB)

Supplementary file21 (JPG 1672 KB)

Supplementary file22 (JPG 1248 KB)

Supplementary file23 (JPG 962 KB)

## Data Availability

All data supporting the findings of this study are available within the article and its Supplementary Information. Additional data are available from the corresponding author upon reasonable request.

## Abbreviations

2D: Two-dimensional
3D: Three-dimensional
α-SMA: Alpha-smooth muscle actin
ANOVA: Analysis of variance
CAD: Computer-aided diagnosis
CCS: Coculture system
COL1: Collagen type I
COL1A1: Collagen type I alpha-1
CO₂: Carbon dioxide
DMEM: Dulbecco’s modified essential medium
EDTA: Ethylenediaminetetraacetic acid
ECM: Extracellular matrix
FACS: Fluorescence-activated cell sorting
FBS: Fetal bovine serum
FITC: Fluorescein isothiocyanate
FSC: Forward scatter
FSC-A: Forward scatter area
FSC-H: Forward scatter height
HCC: Hepatocellular carcinoma
HIF-1α: Hypoxia-inducible factor 1-alpha
HNF4α: Hepatocyte nuclear factor 4 alpha
HSC: Hepatic stellate cells
IC30: 30% Inhibitory concentration
LDH: Lactate dehydrogenase
MFI: Median fluorescence intensity
MGV: Mean gray value
PBS: Phosphate-buffered saline
PI: Propidium iodide
PS: Phosphatidylserine
ROI: Region of interest
Rpm: Rotations per minute
SD: Standard deviation
SEM: Scanning electron microscopy
SEM: Standard error of mean
SSC: Side scatter
SSC-A: Side scatter area
TME: Tumor microenvironmental
UV: Ultraviolet light
