# Supplementary material for: Hepatic stellate cells promote structural and functional remodeling of three-dimensional hepatocellular carcinoma coculture spheroids
Source: Mol Biol Rep. 2026 Sep 12;53(1):1566. doi: 10.1007/s11033-026-12715-9 (PMC13570909; doi:10.1007/s11033-026-12715-9)
Supplement: Supplementary file 1 [file 11033_2026_12715_MOESM20_ESM.docx]

**Hepatic Stellate Cells Promote Structural and Functional Remodeling of Three-Dimensional Hepatocellular Carcinoma Coculture Spheroids**

**Molecular Biology Reports | Springer Nature Link**

Jessica Obelar^1-2^ | João G. Vasconcellos^1^ | Natália Baltazar do Nascimento^1-2^ | Arieli Cruz de Sousa^1-2^ | Maria Giehl^1^ | Benjamim Fischer^1^ | Janaína de Souza Ferreira^1-2^ | Jade de Oliveira ^1-2^ | Vera M. T. Trindade^1-2^ | Fátima T. C. R. Guma^1-2^

^1^ Departamento de Bioquímica, Instituto de Ciências Básicas da Saúde, Universidade Federal do Rio Grande do Sul, Porto Alegre, Rio Grande do Sul, Brazil.

^2^ Programa de Pós-Graduação em Ciências Biológicas: Bioquímica, Instituto de Ciências Básicas da Saúde, Universidade Federal do Rio Grande do Sul, Porto Alegre, Rio Grande do Sul, Brazil.

jessica.obelar@ufrgs.br	https://orcid.org/0000-0003-1213-4519

joaogarciavasc@gmail.com	https://orcid.org/0009-0007-7009-606X

shinataliabn@gmail.com	https://orcid.org/0000-0001-6820-7452

arieli.sousa@ufrgs.br	https://orcid.org/0000-0001-5518-2217

giehlmarialuiza@gmail.com	https://orcid.org/0009-0001-9224-4680
benjamimsfischer@gmail.com	https://orcid.org/0009-0002-4446-4926

janainaferreirasf@gmail.com	https://orcid.org/0009-0003-5935-4890

deoliveirajade10@gmail.com	https://orcid.org/0000-0003-1454-2866

vmtt@ufrgs.br	https://orcid.org/0000-0001-5602-6013

fatima.guma@ufrgs.br	https://orcid.org/0000-0003-2369-7739

**Correspondence:** Jessica Obelar ([jessica.obelar@ufrgs.br](mailto:jessica.obelar@ufrgs.br))

**Supplementary methodology**

*Supplementary Methodology S1. Diameter calculation*

To calculate the diameter of the spheroids we assumed their shapes as circles. The equation for the area of a circle is given by $A=\pi r^{2}$, where $r$ is the radius. Since the diameter of a circle is given by $D= 2r$, we can write $D= 2\sqrt{\frac{A}{\pi}}$, which gives us the diameter of the spheroids in terms of the measured area.

*Supplementary Methodology S2. Sample preparation for flow cytometric immunofluorescence staining*

For each biological replicate, 6–8 spheroids from each experimental group were pooled after 96 h of culture. Three independent biological replicates were analyzed. Spheroids were incubated with TrypLE Express (Thermo Fisher Scientific, USA) for 10 min at room temperature and gently dissociated by mechanical pipetting to obtain single-cell suspensions. The resulting suspensions were centrifuged at 1,200 rpm for 5 min and washed twice with phosphate-buffered saline (PBS). Cells were fixed and permeabilized using the BD Cytofix/Cytoperm™ kit (BD Biosciences, USA) for 20 min at 4 °C, according to the manufacturer’s instructions. Following fixation and permeabilization, cells were washed twice with 1× BD Perm/Wash buffer. No separate blocking step was performed. Primary and secondary antibodies were diluted in 1× BD Perm/Wash buffer. Unless otherwise specified, antibody incubations were performed for 30 min at 4 °C protected from light. All washing steps were followed by centrifugation at 1,200 rpm for 5 min. Because the markers required different primary antibody species and fluorophore combinations, staining was performed in two separate tubes corresponding to two independent antibody panels. For the HNF4α/active caspase-3 panel, cells were incubated with mouse anti-HNF4α antibody (cat. no. MA1-199; Thermo Fisher Scientific, USA; 1:100) and rabbit anti-active caspase-3 antibody (cat. no. C8487; Sigma-Aldrich, USA; 1:10). Following two washes with 1× BD Perm/Wash buffer, cells were incubated with Alexa Fluor® 647-conjugated anti-mouse secondary antibody (cat. no. 4410; Cell Signaling Technology, USA; 1:1000) and Alexa Fluor® 488-conjugated anti-rabbit secondary antibody (cat. no. 4412; Cell Signaling Technology, USA; 1:1000). For the α-SMA/COL1A1 panel, cells were first incubated with rabbit anti-COL1A1 antibody (cat. no. 72026S; Cell Signaling Technology, USA; 1:100). Following two washes with 1× BD Perm/Wash buffer, cells were incubated with Alexa Fluor® 647-conjugated anti-rabbit secondary antibody (cat. no. 4414; Cell Signaling Technology, USA; 1:1000) and directly conjugated Alexa Fluor® 488 anti-α-SMA antibody (cat. no. 53-9760-82; Invitrogen, USA; 1:100).

After staining, samples were washed twice with 1× BD Perm/Wash buffer and resuspended in 100 µL of PBS for flow cytometric acquisition. Unstained controls, single-stained controls for each fluorophore, and secondary-antibody-only controls were included. Single-stained controls were used to calculate and apply the fluorescence compensation matrix in FlowJo version 10. Secondary-antibody-only controls were used to evaluate nonspecific fluorescence and define the negative populations for HNF4α, active caspase-3, and COL1A1. Because fluorescence-minus-one and isotype controls were not included for the directly conjugated α-SMA antibody, α-SMA-positive populations were defined relative to the corresponding unstained control. Data were acquired using a BD Accuri™ C6 flow cytometer (BD Biosciences, USA), with 10,000 events collected per sample. Compensation was not applied during acquisition and was subsequently calculated in FlowJo using the corresponding single-stained controls. Cellular events were initially selected based on forward-scatter area and side-scatter area characteristics to exclude debris. Doublets were then excluded using FSC-H versus FSC-A, and subsequent fluorescence analyses were performed within the singlet-cell population. The complete gating strategy is shown in Supplementary Fig. S1. In the HNF4α/active caspase-3 panel, active caspase-3 fluorescence was evaluated in the total singlet population and separately within the HNF4α-positive and HNF4α-negative subpopulations. In the α-SMA/COL1A1 panel, COL1A1 fluorescence was evaluated in the total singlet population and separately within the α-SMA-positive and α-SMA-negative subpopulations. Data were analyzed using FlowJo version 10 (FlowJo LLC). The percentage of marker-positive cells and median fluorescence intensity (MFI) were determined within the corresponding gates. Corrected median fluorescence intensity was calculated by subtracting the MFI of the matched negative control from the MFI of the stained sample:

$$Corrected MFI=MFI stained sample -MFI matched negative control$$

(1)

For indirectly detected markers, the corresponding secondary-antibody-only control was used as the matched negative control. For directly conjugated α-SMA staining, the unstained control was used.

**Supplementary figure**

**Supplementary Fig. S1 Flow cytometry gating strategy used for immunofluorescence analyses.** Cells were initially selected based on forward scatter (FSC-A) and side scatter (SSC-A) characteristics to exclude debris (left panel). Singlet cells were subsequently identified by FSC-H versus FSC-A gating (right panel). Median fluorescence intensity (MFI) and the percentage of positive cells for HNF4α, active caspase-3, α-SMA, and COL1A1 were determined from the gated singlet population.

**Supplementary Fig. S2 Kinetic determination of intracellular and extracellular lactate dehydrogenase (LDH) activity in HepG2, LX-2, and coculture system (CCS) spheroids.** (a) Intracellular LDH reaction kinetics monitored by the decrease in absorbance at 340 nm over 4 min. (b) Intracellular LDH activity expressed as the rate of absorbance change (ΔA/min). (c) Extracellular LDH reaction kinetics monitored by the decrease in absorbance at 340 nm over 4 min. (d) Extracellular LDH activity expressed as ΔA/min. These kinetic measurements were used to calculate the LDH activity (U/L) presented in the main manuscript. Data are presented as mean ± SD from three independent biological replicates (n = 3), each consisting of four pooled spheroids. Statistical significance was determined by one-way ANOVA followed by Tukey's multiple comparisons test. Exact *p* values are indicated in the figure.

**Supplementary Fig. S3. Flow cytometric characterization of the cellular composition of coculture system (CCS) spheroids using HNF4α expression.** (a) Representative dot plots showing HNF4α immunofluorescence (Alexa Fluor® 647; FL4-A channel) in unstained cells, LX-2 monocultures, HepG2 monocultures, and CCS spheroids. (b) Representative density plot of CCS spheroids showing the discrimination of HNF4α-positive (HepG2-like) and HNF4α-negative (LX-2-like) cell populations. (c) Representative histograms of HNF4α fluorescence in unstained cells, CCS spheroids, and the HNF4α-positive and HNF4α-negative populations identified within CCS. The dashed line indicates the threshold for HNF4α positivity established using the unstained control. (d) Quantification of the cellular composition of CCS spheroids based on HNF4α expression, showing the percentage of HNF4α-positive (HepG2-like) and HNF4α-negative (LX-2-like) cells. Data are presented as mean ± SD from three independent biological replicates (n = 3), each consisting of 6–8 pooled spheroids. Statistical significance was determined using an unpaired Student's t-test. Exact p values are indicated in the figure.

**Supplementary Fig. S4** **Relative spheroid growth after 24 hours in monoculture and co-culture systems.** Spheroid growth was assessed after 24 hours in HepG2, LX-2, and co-culture spheroids (CCS), and expressed as relative growth normalized to HepG2. No statistically significant differences were observed between the groups (HepG2 vs. LX-2, p = 0.9383; HepG2 vs. CCS, p = 0.2794; LX-2 vs. CCS, p = 0.4429). Data are presented as mean ± SD.

**Supplementary Fig. S5. Temporal analysis of spheroid diameter in monoculture and co-culture systems.** Spheroids derived from HepG2 (blue dashed line), LX-2 (gray dashed line), and co-culture (CCS; black solid line) were measured at 2, 24, 48, 72, and 96 hours. HepG2 spheroids showed a rapid increase in diameter after 2 h, maintaining a larger size throughout the experiment, while CCS spheroids exhibited a progressive reduction in diameter over time. Data are expressed as mean ± SD. Blue asterisks indicate statistical differences between CCS and HepG2 (*p < 0.05; **** p < 0.0001). Blue hashtags indicate statistical differences between CCS and LX-2 (# p < 0.05; #### p < 0.0001).

**Supplementary tables**

**Supplementary Table S1. Antibodies used for flow cytometric immunofluorescence staining.**

| **Target/reagent** | **Antibody role** | **Fluorophore** | **Manufacturer** | **Catalog no.** | **Working dilution** |
| --- | --- | --- | --- | --- | --- |
| HNF4α | 1^ary^ antibody | - | Thermo Fisher Scientific | MA1-199 | 1:100 |
| Active caspase-3 | 1^ary^ antibody | - | Sigma-Aldrich | C8487 | 1:10 |
| COL1A1 | 1^ary^ antibody | - | Cell Signaling Technology | 72026S | 1:100 |
| α-SMA | 1^ary^ antibody | Alexa Fluor 488 | Invitrogen | 53-9760-82 | 1:100 |
| Anti-mouse secondary | 2^ary^ antibody | Alexa Fluor 647 | Cell Signaling Technology | 4410 | 1:1000 |
| Anti-rabbit secondary | 2^ary^ antibody | Alexa Fluor 488 | Cell Signaling Technology | 4412 | 1:1000 |
| Anti-rabbit secondary | 2^ary^ antibody | Alexa Fluor 647 | Cell Signaling Technology | 4414 | 1:1000 |

**Supplementary Table S2. Pairwise comparisons of spheroid projected area among culture models.** Mean projected spheroid area ± SEM is presented for HepG2, LX-2, and CCS spheroids at 2, 24, 48, 72 and 96 h. Relative differences (%) were calculated as [(Mean 2−Mean 1)/Mean 1]×100, with the first model in each comparison used as the reference. Positive values indicate that Model 2 had a larger mean area than Model 1, whereas negative values indicate a smaller mean area. Statistical analysis was performed using two-way ANOVA, followed by Tukey’s multiple-comparisons test to compare culture models within each time point. *p < 0.05 indicates statistical significance.

| **Area** | Mean 1 (mm²) | SEM1 | Mean 2 (mm²) | SEM2 | Relative change (%) | P-value |
| --- | --- | --- | --- | --- | --- | --- |
| **2 h** |  |  |  |  |  |  |
| CCS vs. HepG2 | 1.303 | 0.1224 | 1.718 | 0.27 | 31.85 | 0.0374* |
| CCS vs. LX-2 | 1.303 | 0.1224 | 1.419 | 0.2607 | 8.90 | 0.6692 |
| HepG2 vs. LX-2 | 1.718 | 0.27 | 1.419 | 0.2607 | -17.40 | 0.1724 |
| **24 h** |  |  |  |  |  |  |
| CCS vs. HepG2 | 0.3693 | 0.03048 | 1.354 | 0.1088 | 266.64 | <0.0001* |
| CCS vs. LX-2 | 0.3693 | 0.03048 | 0.3973 | 0.02061 | 7.58 | 0.9765 |
| HepG2 vs. LX-2 | 1.354 | 0.1088 | 0.3973 | 0.02061 | -70.66 | <0.0001* |
| **48 h** |  |  |  |  |  |  |
| CCS vs. HepG2 | 0.33 | 0.0291 | 1.523 | 0.0965 | 361.51 | <0.0001* |
| CCS vs. LX-2 | 0.33 | 0.0291 | 0.314 | 0.02833 | -4.85 | 0.9923 |
| HepG2 vs. LX-2 | 1.523 | 0.0965 | 0.314 | 0.02833 | -79.38 | <0.0001* |
| **72 h** |  |  |  |  |  |  |
| CCS vs. HepG2 | 0.2863 | 0.02963 | 1.335 | 0.03239 | 366.29 | <0.0001* |
| CCS vs. LX-2 | 0.2863 | 0.02963 | 0.2878 | 0.02656 | 0.52 | >0.9999 |
| HepG2 vs. LX-2 | 1.335 | 0.03239 | 0.2878 | 0.02656 | -78.44 | <0.0001* |
| **96 h** |  |  |  |  |  |  |
| CCS vs. HepG2 | 0.2882 | 0.02873 | 1.341 | 0.06086 | 365.30 | <0.0001* |
| CCS vs. LX-2 | 0.2882 | 0.02873 | 0.2778 | 0.02299 | -3.61 | 0.9968 |
| HepG2 vs. LX-2 | 1.341 | 0.06086 | 0.2778 | 0.02299 | -79.28 | <0.0001* |

**Supplementary Table S3. Temporal changes in spheroid area within each coculture model.** Mean projected spheroid area ± SEM is presented for HepG2, LX-2 and CCS models at 2, 24, 48, 72, and 96 h. Each subsequent time point was compared with the corresponding 2-h value. Relative change was calculated as [(area at the comparison time−area at 2 h)/area at 2 h]×100. Negative values indicate a reduction in projected spheroid area relative to 2 h. Statistical analysis was performed using two-way ANOVA followed by Tukey’s multiple-comparisons test. *p < 0.05 indicates statistical significance.

| **Area** | Mean 1 (mm²) | SEM1 | Mean 2 (mm²) | SEM2 | Relative change (%) | P-value |
| --- | --- | --- | --- | --- | --- | --- |
| **HepG2** |  |  |  |  |  |  |
| 2 h vs. 24 h | 1.718 | 0.27 | 1.354 | 0.11 | -21.19 | 0.1165 |
| 2 h vs. 48 h | 1.718 | 0.27 | 1.523 | 0.10 | -11.35 | 0.5530 |
| 2 h vs. 72 h | 1.718 | 0.27 | 1.335 | 0.03 | -22.29 | 0.1084 |
| 2 h vs. 96 h | 1.718 | 0.27 | 1.341 | 0.06 | -21.94 | 0.1002 |
| **LX-2** |  |  |  |  |  |  |
| 2 h vs. 24 h | 1.419 | 0.26 | 0.3973 | 0.02 | -72.00 | <0.0001* |
| 2 h vs. 48 h | 1.419 | 0.26 | 0.314 | 0.03 | -77.87 | <0.0001* |
| 2 h vs. 72 h | 1.419 | 0.26 | 0.2878 | 0.03 | -79.72 | <0.0001* |
| 2 h vs. 96 h | 1.419 | 0.26 | 0.2778 | 0.02 | -80.42 | <0.0001* |
| **CCS** |  |  |  |  |  |  |
| 2 h vs. 24 h | 1.303 | 0.12 | 0.3693 | 0.03 | -71.66 | <0.0001* |
| 2 h vs. 48 h | 1.303 | 0.12 | 0.33 | 0.03 | -74.67 | <0.0001* |
| 2 h vs. 72 h | 1.303 | 0.12 | 0.2863 | 0.03 | -78.03 | <0.0001* |
| 2 h vs. 96 h | 1.303 | 0.12 | 0.2882 | 0.03 | -77.88 | <0.0001* |

**Supplementary Table S4. Temporal changes in spheroid perimeter within each coculture model.** Mean perimeter ± SEM is presented for HepG2, LX-2 and CCS models at 2, 24, 48, 72, and 96 h. Each subsequent time point was compared with the corresponding 2-h value. Relative change was calculated as [(perimeter at the comparison time−perimeter at 2 h)/area at 2 h]×100. Negative values indicate a reduction in spheroid perimeter relative to 2 h. Statistical analysis was performed using two-way ANOVA followed by Tukey’s multiple-comparisons test. *p < 0.05 indicates statistical significance.

| **Perimeter** | Mean 1 (mm²) | SEM1 | Mean 2 (mm²) | SEM2 | Relative change (%) | P-value |
| --- | --- | --- | --- | --- | --- | --- |
| **HepG2** |  |  |  |  |  |  |
| 2 h vs. 24 h | 17.230 | 4.690 | 12.600 | 1.230 | -26.87 | 0.398 |
| 2 h vs. 48 h | 17.230 | 4.690 | 9.710 | 0.650 | -43.64 | 0.905 |
| 2 h vs. 72 h | 17.230 | 4.690 | 12.900 | 2.270 | -25.13 | 0.450 |
| 2 h vs. 96 h | 17.230 | 4.690 | 13.130 | 2.140 | -23.79 | 0.494 |
| **LX-2** |  |  |  |  |  |  |
| 2 h vs. 24 h | 11.900 | 1.861 | 4.335 | 0.553 | -63.57 | <0.0001* |
| 2 h vs. 48 h | 11.900 | 1.861 | 3.187 | 0.418 | -73.22 | <0.0001* |
| 2 h vs. 72 h | 11.900 | 1.861 | 3.530 | 0.681 | -70.34 | <0.0001* |
| 2 h vs. 96 h | 11.900 | 1.861 | 2.732 | 0.170 | -77.04 | <0.0001* |
| **CCS** |  |  |  |  |  |  |
| 2 h vs. 24 h | 11.950 | 1.460 | 3.961 | 0.402 | -66.85 | <0.0001* |
| 2 h vs. 48 h | 11.950 | 1.460 | 3.922 | 0.511 | -67.18 | <0.0001* |
| 2 h vs. 72 h | 11.950 | 1.460 | 3.619 | 0.804 | -69.71 | <0.0001* |
| 2 h vs. 96 h | 11.950 | 1.460 | 3.487 | 0.609 | -70.82 | <0.0001* |

**Supplementary Table S5. Pairwise comparisons of spheroid perimeter among culture models.** Mean projected spheroid perimeter ± SEM are presented for HepG2, LX-2, and CCS spheroids at 2, 24, 48, 72 and 96 h. Relative differences (%) were calculated as [(Mean 2−Mean 1)/Mean 1]×100, with the first model in each comparison used as the reference. Positive values indicate that Model 2 had a larger mean perimeter than Model 1, whereas negative values indicate a smaller mean perimeter. Statistical analysis was performed using two-way ANOVA, followed by Tukey’s multiple-comparisons test to compare culture models within each time point. *p < 0.05 indicates statistical significance.

| **Perimeter** | Mean 1 (mm²) | SEM1 | Mean 2 (mm²) | SEM2 | Relative change (%) | P-value |
| --- | --- | --- | --- | --- | --- | --- |
| **2 h** |  |  |  |  |  |  |
| CCS vs. HepG2 | 11.950 | 1.460 | 17.230 | 4.690 | 44.18 | 0.0567 |
| CCS vs. LX-2 | 11.950 | 1.460 | 11.900 | 1.861 | -0.42 | 0.9995 |
| HepG2 vs. LX-2 | 17.230 | 4.690 | 11.900 | 1.861 | -30.93 | 0.0537 |
| **24 h** |  |  |  |  |  |  |
| CCS vs. HepG2 | 3.961 | 0.402 | 12.600 | 1.230 | 218.10 | <0.0001* |
| CCS vs. LX-2 | 3.961 | 0.402 | 4.335 | 0.553 | 9.44 | 0.9775 |
| HepG2 vs. LX-2 | 12.600 | 1.230 | 4.335 | 0.553 | -65.60 | <0.0001* |
| **48 h** |  |  |  |  |  |  |
| CCS vs. HepG2 | 3.922 | 0.511 | 9.710 | 0.650 | 147.58 | 0.0068* |
| CCS vs. LX-2 | 3.922 | 0.511 | 3.187 | 0.418 | -18.74 | 0.9162 |
| HepG2 vs. LX-2 | 9.710 | 0.650 | 3.187 | 0.418 | -67.19 | 0.0020* |
| **72 h** |  |  |  |  |  |  |
| CCS vs. HepG2 | 3.619 | 0.804 | 12.900 | 2.270 | 256.45 | <0.0001* |
| CCS vs. LX-2 | 3.619 | 0.804 | 3.530 | 0.681 | -2.46 | 0.9987 |
| HepG2 vs. LX-2 | 12.900 | 2.270 | 3.530 | 0.681 | -72.64 | <0.0001* |
| **96 h** |  |  |  |  |  |  |
| CCS vs. HepG2 | 3.487 | 0.609 | 13.130 | 2.140 | 276.54 | <0.0001* |
| CCS vs. LX-2 | 3.487 | 0.609 | 2.732 | 0.170 | -21.65 | 0.9194 |
| HepG2 vs. LX-2 | 13.130 | 2.140 | 2.732 | 0.170 | -79.19 | <0.0001* |

**Supplementary Table S6.Pairwise comparisons of spheroid circularity among culture models.** Mean spheroid circularity ± SEM is presented for HepG2, LX-2, and CCS spheroids at 2, 24, 48, 72, and 96 h. Relative differences (%) were calculated as [(Mean 2−Mean 1)/Mean 1]×100[(Mean\ 2-Mean\ 1)/Mean\ 1]\times100[(Mean 2−Mean 1)/Mean 1]×100, with the first model in each comparison used as the reference. Positive values indicate that Model 2 had greater mean circularity than Model 1, whereas negative values indicate lower mean circularity. Statistical analysis was performed using two-way ANOVA followed by Tukey’s multiple-comparisons test to compare culture models within each time point. *p < 0.05 indicates statistical significance.

| **Circularity** | Mean 1 | SEM1 | Mean 2 | SEM2 | Relative change (%) | P-value |
| --- | --- | --- | --- | --- | --- | --- |
| **2 h** |  |  |  |  |  |  |
| CCS vs. HepG2 | 0.105 | 0.021 | 0.115 | 0.067 | 9.39 | 0.9966 |
| CCS vs. LX-2 | 0.105 | 0.021 | 0.148 | 0.038 | 40.13 | 0.9150 |
| HepG2 vs. LX-2 | 0.115 | 0.067 | 0.148 | 0.038 | 28.10 | 0.9626 |
| **24 h** |  |  |  |  |  |  |
| CCS vs. HepG2 | 0.323 | 0.048 | 0.122 | 0.024 | -62.19 | 0.1196 |
| CCS vs. LX-2 | 0.323 | 0.048 | 0.346 | 0.095 | 7.13 | 0.9715 |
| HepG2 vs. LX-2 | 0.122 | 0.024 | 0.346 | 0.095 | 183.36 | 0.0731 |
| **48 h** |  |  |  |  |  |  |
| CCS vs. HepG2 | 0.337 | 0.082 | 0.217 | 0.030 | -35.80 | 0.4555 |
| CCS vs. LX-2 | 0.337 | 0.082 | 0.447 | 0.077 | 32.56 | 0.5204 |
| HepG2 vs. LX-2 | 0.217 | 0.030 | 0.447 | 0.077 | 106.47 | 0.0626 |
| **72 h** |  |  |  |  |  |  |
| CCS vs. HepG2 | 0.435 | 0.112 | 0.160 | 0.050 | -63.24 | 0.0210* |
| CCS vs. LX-2 | 0.435 | 0.112 | 0.398 | 0.992 | -8.37 | 0.9303 |
| HepG2 vs. LX-2 | 0.160 | 0.050 | 0.398 | 0.992 | 149.25 | 0.0520 |
| **96 h** |  |  |  |  |  |  |
| CCS vs. HepG2 | 0.411 | 0.113 | 0.137 | 0.036 | -66.61 | 0.0215* |
| CCS vs. LX-2 | 0.411 | 0.113 | 0.405 | 0.067 | -1.63 | 0.9976 |
| HepG2 vs. LX-2 | 0.137 | 0.036 | 0.405 | 0.067 | 194.61 | 0.0256* |

**Supplementary Table S7.** **Temporal changes in spheroid circularity within each culture model.** Mean spheroid circularity ± SEM is presented for HepG2, LX-2, and CCS spheroids at 2, 24, 48, 72, and 96 h. Each subsequent time point was compared with the corresponding 2-h value. Relative change was calculated as [(circularity at the comparison time − circularity at 2 h)/circularity at 2 h] × 100. Positive values indicate an increase, whereas negative values indicate a reduction in spheroid circularity relative to 2 h. Statistical analysis was performed using two-way ANOVA followed by Tukey’s multiple-comparisons test. *p < 0.05 indicates statistical significance.

| **Circularity** | Mean 1 | SEM1 | Mean 2 | SEM2 | Relative change (%) | P-value |
| --- | --- | --- | --- | --- | --- | --- |
| **HepG2** |  |  |  |  |  |  |
| 2 h vs. 24 h | 0.115 | 0.067 | 0.122 | 0.024 | 5.81 | 0.99980 |
| 2 h vs. 48 h | 0.115 | 0.067 | 0.217 | 0.030 | 87.77 | 0.31450 |
| 2 h vs. 72 h | 0.115 | 0.067 | 0.160 | 0.050 | 38.60 | 0.85970 |
| 2 h vs. 96 h | 0.115 | 0.067 | 0.137 | 0.036 | 19.08 | 0.98580 |
| **LX-2** |  |  |  |  |  |  |
| 2 h vs. 24 h | 0.148 | 0.038 | 0.346 | 0.095 | 134.06 | 0.24850 |
| 2 h vs. 48 h | 0.148 | 0.038 | 0.447 | 0.077 | 202.64 | 0.0414* |
| 2 h vs. 72 h | 0.148 | 0.038 | 0.398 | 0.992 | 169.67 | 0.10360 |
| 2 h vs. 96 h | 0.148 | 0.038 | 0.405 | 0.067 | 173.87 | 0.09270 |
| **CCS** |  |  |  |  |  |  |
| 2 h vs. 24 h | 0.105 | 0.021 | 0.323 | 0.048 | 206.17 | 0.26320 |
| 2 h vs. 48 h | 0.105 | 0.021 | 0.337 | 0.082 | 219.92 | 0.21660 |
| 2 h vs. 72 h | 0.105 | 0.021 | 0.435 | 0.112 | 312.43 | 0.0477* |
| 2 h vs. 96 h | 0.105 | 0.021 | 0.411 | 0.113 | 290.13 | 0.07060 |

**Supplementary Table S8. Temporal changes in spheroid roundness within each culture model.** Mean spheroid roundness ± SEM is presented for HepG2, LX-2, and CCS spheroids at 2, 24, 48, 72, and 96 h. Each subsequent time point was compared with the corresponding 2-h value. Relative change was calculated as [(roundness at the comparison time − roundness at 2 h)/roundness at 2 h] × 100. Positive values indicate an increase, whereas negative values indicate a reduction in spheroid roundness relative to 2 h. Statistical analysis was performed using two-way ANOVA followed by Tukey’s multiple-comparisons test. *p < 0.05 indicates statistical significance.

| **Roundness** | Mean 1 | SEM1 | Mean 2 | SEM2 | Relative change (%) | P-value |
| --- | --- | --- | --- | --- | --- | --- |
| **HepG2** |  |  |  |  |  |  |
| 2 h vs. 24 h | 0.827 | 0.085 | 0.849 | 0.020 | 2.67 | 0.91960 |
| 2 h vs. 48 h | 0.827 | 0.085 | 0.864 | 0.011 | 4.45 | 0.69500 |
| 2 h vs. 72 h | 0.827 | 0.085 | 0.840 | 0.011 | 1.65 | 0.98420 |
| 2 h vs. 96 h | 0.827 | 0.085 | 0.843 | 0.013 | 1.91 | 0.97330 |
| **LX-2** |  |  |  |  |  |  |
| 2 h vs. 24 h | 0.547 | 0.096 | 0.706 | 0.047 | 29.14 | 0.08860 |
| 2 h vs. 48 h | 0.547 | 0.096 | 0.851 | 0.016 | 55.73 | 0.00050* |
| 2 h vs. 72 h | 0.547 | 0.096 | 0.880 | 0.011 | 60.91 | <0.0001* |
| 2 h vs. 96 h | 0.547 | 0.096 | 0.894 | 0.008 | 63.53 | <0.0001* |
| **CCS** |  |  |  |  |  |  |
| 2 h vs. 24 h | 0.690 | 0.042 | 0.607 | 0.144 | -12.03 | 0.97020 |
| 2 h vs. 48 h | 0.690 | 0.042 | 0.650 | 0.135 | -5.75 | 0.99760 |
| 2 h vs. 72 h | 0.690 | 0.042 | 0.611 | 0.174 | -11.49 | 0.96990 |
| 2 h vs. 96 h | 0.690 | 0.042 | 0.847 | 0.052 | 22.80 | 0.75470 |

**Supplementary Table S9. Pairwise comparisons of spheroid roundness among culture models.** Mean spheroid roundness ± SEM is presented for HepG2, LX-2, and CCS spheroids at 2, 24, 48, 72, and 96 h. Relative differences (%) were calculated as [(Mean 2−Mean 1)/Mean 1]×100, with the first model in each comparison used as the reference. Positive values indicate that Model 2 had greater mean roundness than Model 1, whereas negative values indicate lower mean roundness. Statistical analysis was performed using two-way ANOVA followed by Tukey’s multiple-comparisons test to compare culture models within each time point. *p < 0.05 indicates statistical significance.

| **Roundness** | Mean 1 | SEM1 | Mean 2 | SEM2 | Relative change (%) | P-value |
| --- | --- | --- | --- | --- | --- | --- |
| **2 h** |  |  |  |  |  |  |
| CCS vs. HepG2 | 0.690 | 0.042 | 0.827 | 0.085 | 19.81 | 0.6061 |
| CCS vs. LX-2 | 0.690 | 0.042 | 0.547 | 0.096 | -20.77 | 0.4398 |
| HepG2 vs. LX-2 | 0.827 | 0.085 | 0.547 | 0.096 | -33.87 | 0.1295 |
| **24 h** |  |  |  |  |  |  |
| CCS vs. HepG2 | 0.607 | 0.144 | 0.849 | 0.020 | 39.84 | 0.1251 |
| CCS vs. LX-2 | 0.607 | 0.144 | 0.706 | 0.047 | 16.31 | 0.6982 |
| HepG2 vs. LX-2 | 0.849 | 0.020 | 0.706 | 0.047 | -16.82 | 0.4423 |
| **48 h** |  |  |  |  |  |  |
| CCS vs. HepG2 | 0.650 | 0.135 | 0.864 | 0.011 | 32.79 | 0.1677 |
| CCS vs. LX-2 | 0.650 | 0.135 | 0.864 | 0.011 | 32.79 | 0.2339 |
| HepG2 vs. LX-2 | 0.864 | 0.011 | 0.851 | 0.016 | -1.40 | 0.9946 |
| **72 h** |  |  |  |  |  |  |
| CCS vs. HepG2 | 0.611 | 0.174 | 0.840 | 0.011 | 37.60 | 0.1271 |
| CCS vs. LX-2 | 0.611 | 0.174 | 0.840 | 0.011 | 37.60 | 0.0612 |
| HepG2 vs. LX-2 | 0.840 | 0.011 | 0.880 | 0.011 | 4.69 | 0.9392 |
| **96 h** |  |  |  |  |  |  |
| CCS vs. HepG2 | 0.847 | 0.052 | 0.843 | 0.013 | -0.57 | 0.9991 |
| CCS vs. LX-2 | 0.847 | 0.052 | 0.843 | 0.013 | -0.57 | 0.9155 |
| HepG2 vs. LX-2 | 0.843 | 0.013 | 0.894 | 0.008 | 6.11 | 0.8981 |

**Supplementary Table S10. Temporal changes in spheroid aspect ratio within each culture model.** Mean spheroid aspect ratio ± SEM is presented for HepG2, LX-2, and CCS spheroids at 2, 24, 48, 72, and 96 h. Each subsequent time point was compared with the corresponding 2-h value. Relative change was calculated as [(aspect ratio at the comparison time − aspect ratio at 2 h)/aspect ratio at 2 h] × 100. Positive values indicate an increase, whereas negative values indicate a reduction in spheroid aspect ratio relative to 2 h. Statistical analysis was performed using two-way ANOVA followed by Tukey’s multiple-comparisons test. *p < 0.05 indicates statistical significance.

| **Aspect ratio** | Mean 1 | SEM1 | Mean 2 | SEM2 | Relative change (%) | P-value |
| --- | --- | --- | --- | --- | --- | --- |
| **HepG2** |  |  |  |  |  |  |
| 2 h vs. 24 h | 1.239 | 0.143 | 1.181 | 0.027 | -4.68 | 0.70480 |
| 2 h vs. 48 h | 1.239 | 0.143 | 1.159 | 0.016 | -6.46 | 0.46310 |
| 2 h vs. 72 h | 1.239 | 0.143 | 1.191 | 0.016 | -3.87 | 0.81050 |
| 2 h vs. 96 h | 1.239 | 0.143 | 1.188 | 0.017 | -4.12 | 0.78110 |
| **LX-2** |  |  |  |  |  |  |
| 2 h vs. 24 h | 1.349 | 0.134 | 1.495 | 0.160 | 10.82 | 0.66900 |
| 2 h vs. 48 h | 1.349 | 0.134 | 1.239 | 0.066 | -8.15 | 0.83310 |
| 2 h vs. 72 h | 1.349 | 0.134 | 1.139 | 0.021 | -15.58 | 0.37320 |
| 2 h vs. 96 h | 1.349 | 0.134 | 1.120 | 0.016 | -16.98 | 0.30230 |
| **CCS** |  |  |  |  |  |  |
| 2 h vs. 24 h | 1.478 | 0.092 | 1.206 | 0.044 | -18.40 | 0.08880 |
| 2 h vs. 48 h | 1.478 | 0.092 | 1.186 | 0.066 | -19.76 | 0.06230 |
| 2 h vs. 72 h | 1.478 | 0.092 | 1.224 | 0.094 | -17.19 | 0.09790 |
| 2 h vs. 96 h | 1.478 | 0.092 | 1.204 | 0.080 | -18.54 | 0.06790 |

**Supplementary Table S11. Pairwise comparisons of spheroid aspect ratio among culture models.** Mean spheroid aspect ratio ± SEM is presented for HepG2, LX-2, and CCS spheroids at 2, 24, 48, 72, and 96 h. Relative differences (%) were calculated as [(Mean 2 − Mean 1)/Mean 1] × 100, with the first model in each comparison used as the reference. Positive values indicate that Model 2 had a greater mean aspect ratio than Model 1, whereas negative values indicate a lower mean aspect ratio. Statistical analysis was performed using two-way ANOVA followed by Tukey’s multiple-comparisons test to compare culture models within each time point. *p < 0.05 indicates statistical significance.

| **Aspect ratio** | Mean 1 | SEM1 | Mean 2 | SEM2 | Relative change (%) | P-value |
| --- | --- | --- | --- | --- | --- | --- |
| **2 h** |  |  |  |  |  |  |
| CCS vs. HepG2 | 1.478 | 0.092 | 1.239 | 0.143 | -16.17 | 0.1581 |
| CCS vs. LX-2 | 1.478 | 0.092 | 1.349 | 0.134 | -8.73 | 0.4746 |
| HepG2 vs. LX-2 | 1.239 | 0.143 | 1.349 | 0.134 | 8.89 | 0.6854 |
| **24 h** |  |  |  |  |  |  |
| CCS vs. HepG2 | 1.206 | 0.044 | 1.181 | 0.027 | -2.07 | 0.9722 |
| CCS vs. LX-2 | 1.206 | 0.044 | 1.495 | 0.160 | 23.96 | 0.0286* |
| HepG2 vs. LX-2 | 1.181 | 0.027 | 1.495 | 0.160 | 26.59 | 0.0107* |
| **48 h** |  |  |  |  |  |  |
| CCS vs. HepG2 | 1.186 | 0.066 | 1.159 | 0.016 | -2.28 | 0.9668 |
| CCS vs. LX-2 | 1.186 | 0.066 | 1.239 | 0.066 | 4.47 | 0.8830 |
| HepG2 vs. LX-2 | 1.159 | 0.016 | 1.239 | 0.066 | 6.90 | 0.7296 |
| **72 h** |  |  |  |  |  |  |
| CCS vs. HepG2 | 1.224 | 0.094 | 1.191 | 0.016 | -2.70 | 0.9469 |
| CCS vs. LX-2 | 1.224 | 0.094 | 1.139 | 0.021 | -6.94 | 0.6956 |
| HepG2 vs. LX-2 | 1.191 | 0.016 | 1.139 | 0.021 | -4.37 | 0.8719 |
| **96 h** |  |  |  |  |  |  |
| CCS vs. HepG2 | 1.204 | 0.080 | 1.188 | 0.017 | -1.33 | 0.9872 |
| CCS vs. LX-2 | 1.204 | 0.080 | 1.120 | 0.016 | -6.98 | 0.6996 |
| HepG2 vs. LX-2 | 1.188 | 0.017 | 1.120 | 0.016 | -5.72 | 0.7901 |

**Supplementary Table S12. Pairwise comparisons of spheroid geometric solidity among culture models.** Mean spheroid geometric solidity ± SEM is presented for HepG2, LX-2, and CCS spheroids at 2, 24, 48, 72, and 96 h. Relative differences (%) were calculated as [(Mean 2 − Mean 1)/Mean 1] × 100, with the first model in each comparison used as the reference. Positive values indicate that Model 2 had greater mean geometric solidity than Model 1, whereas negative values indicate lower mean geometric solidity. Statistical analysis was performed using two-way ANOVA followed by Tukey’s multiple-comparisons test to compare culture models within each time point. p < 0.05 indicates statistical significance.

| **Geometric solidity** | Mean 1 | SEM1 | Mean 2 | SEM2 | Relative change (%) | P-value |
| --- | --- | --- | --- | --- | --- | --- |
| **2 h** |  |  |  |  |  |  |
| CCS vs. HepG2 | 0.737 | 0.068 | 0.561 | 0.196 | -23.79 | 0.1950 |
| CCS vs. LX-2 | 0.737 | 0.068 | 0.684 | 0.063 | -7.09 | 0.8304 |
| HepG2 vs. LX-2 | 0.561 | 0.196 | 0.684 | 0.063 | 21.91 | 0.4412 |
| **24 h** |  |  |  |  |  |  |
| CCS vs. HepG2 | 0.869 | 0.050 | 0.636 | 0.134 | -26.82 | 0.0303 |
| CCS vs. LX-2 | 0.869 | 0.050 | 0.786 | 0.068 | -9.53 | 0.6274 |
| HepG2 vs. LX-2 | 0.636 | 0.134 | 0.786 | 0.068 | 23.62 | 0.2222 |
| **48 h** |  |  |  |  |  |  |
| CCS vs. HepG2 | 0.871 | 0.041 | 0.824 | 0.024 | -5.45 | 0.8571 |
| CCS vs. LX-2 | 0.871 | 0.041 | 0.882 | 0.032 | 1.26 | 0.9917 |
| HepG2 vs. LX-2 | 0.824 | 0.024 | 0.882 | 0.032 | 7.10 | 0.7918 |
| **72 h** |  |  |  |  |  |  |
| CCS vs. HepG2 | 0.885 | 0.042 | 0.800 | 0.026 | -9.59 | 0.6134 |
| CCS vs. LX-2 | 0.885 | 0.042 | 0.931 | 0.013 | 5.30 | 0.8723 |
| HepG2 vs. LX-2 | 0.800 | 0.026 | 0.931 | 0.013 | 16.47 | 0.3463 |
| **96 h** |  |  |  |  |  |  |
| CCS vs. HepG2 | 0.898 | 0.038 | 0.807 | 0.026 | -10.21 | 0.5656 |
| CCS vs. LX-2 | 0.898 | 0.038 | 0.936 | 0.012 | 4.21 | 0.9148 |
| HepG2 vs. LX-2 | 0.807 | 0.026 | 0.936 | 0.012 | 16.06 | 0.3586 |

**Supplementary Table S13. Temporal changes in spheroid geometric solidity within each culture model**. Mean spheroid geometric solidity ± SEM is presented for HepG2, LX-2, and CCS spheroids at 2, 24, 48, 72, and 96 h. Each subsequent time point was compared with the corresponding 2-h value. Relative change was calculated as [(geometric solidity at the comparison time − geometric solidity at 2 h)/geometric solidity at 2 h] × 100. Positive values indicate an increase, whereas negative values indicate a reduction in spheroid geometric solidity relative to 2 h. Statistical analysis was performed using two-way ANOVA followed by Tukey’s multiple-comparisons test. p < 0.05 indicates statistical significance.

| **Geometric solidity** | Mean 1 | SEM1 | Mean 2 | SEM2 | Relative change (%) | P-value |
| --- | --- | --- | --- | --- | --- | --- |
| **HepG2** |  |  |  |  |  |  |
| 2 h vs. 24 h | 0.561 | 0.196 | 0.636 | 0.134 | 13.27 | 0.94190 |
| 2 h vs. 48 h | 0.561 | 0.196 | 0.824 | 0.024 | 46.77 | 0.19090 |
| 2 h vs. 72 h | 0.561 | 0.196 | 0.800 | 0.026 | 42.47 | 0.25650 |
| 2 h vs. 96 h | 0.561 | 0.196 | 0.807 | 0.026 | 43.68 | 0.23640 |
| **LX-2** |  |  |  |  |  |  |
| 2 h vs. 24 h | 0.684 | 0.063 | 0.786 | 0.068 | 14.86 | 0.36330 |
| 2 h vs. 48 h | 0.684 | 0.063 | 0.882 | 0.032 | 28.94 | 0.02050 |
| 2 h vs. 72 h | 0.684 | 0.063 | 0.931 | 0.013 | 36.11 | 0.00530 |
| 2 h vs. 96 h | 0.684 | 0.063 | 0.936 | 0.012 | 36.78 | 0.00450 |
| **CCS** |  |  |  |  |  |  |
| 2 h vs. 24 h | 0.737 | 0.068 | 0.869 | 0.050 | 17.96 | 0.20010 |
| 2 h vs. 48 h | 0.737 | 0.068 | 0.871 | 0.041 | 18.30 | 0.18760 |
| 2 h vs. 72 h | 0.737 | 0.068 | 0.885 | 0.042 | 20.10 | 0.13180 |
| 2 h vs. 96 h | 0.737 | 0.068 | 0.898 | 0.038 | 21.96 | 0.08940 |

**Supplementary Table S14. Pairwise comparisons of the MGV-derived spheroid compaction index among culture models.** Mean MGV-derived compaction index ± SEM is presented for HepG2, LX-2, and CCS spheroids at 2, 24, 48, 72, and 96 h. Relative differences (%) were calculated as [(Mean 2 − Mean 1)/Mean 1] × 100, with the first model in each comparison used as the reference. Positive values indicate that Model 2 had a greater mean MGV-derived compaction index than Model 1, whereas negative values indicate a lower mean MGV-derived compaction index. Statistical analysis was performed using two-way ANOVA followed by Tukey’s multiple-comparisons test to compare culture models within each time point. p < 0.05 indicates statistical significance.

| **MGV-derived spheroid compaction index** | Mean 1 | SEM1 | Mean 2 | SEM2 | Relative change (%) | P-value |
| --- | --- | --- | --- | --- | --- | --- |
| **2 h** |  |  |  |  |  |  |
| CCS vs. HepG2 | 0.406 | 0.044 | 0.356 | 0.091 | -12.50 | 0.7634 |
| CCS vs. LX-2 | 0.406 | 0.044 | 0.326 | 0.067 | -19.84 | 0.3655 |
| HepG2 vs. LX-2 | 0.356 | 0.091 | 0.326 | 0.067 | -8.38 | 0.9109 |
| **24 h** |  |  |  |  |  |  |
| CCS vs. HepG2 | 0.772 | 0.029 | 0.514 | 0.034 | -33.41 | 0.0001* |
| CCS vs. LX-2 | 0.772 | 0.029 | 0.660 | 0.022 | -14.54 | 0.1460 |
| HepG2 vs. LX-2 | 0.514 | 0.034 | 0.660 | 0.022 | 28.33 | 0.0421* |
| **48 h** |  |  |  |  |  |  |
| CCS vs. HepG2 | 0.840 | 0.029 | 0.551 | 0.029 | -34.46 | <0.0001* |
| CCS vs. LX-2 | 0.840 | 0.029 | 0.823 | 0.036 | -1.99 | 0.9569 |
| HepG2 vs. LX-2 | 0.551 | 0.029 | 0.823 | 0.036 | 49.55 | <0.0001* |
| **72 h** |  |  |  |  |  |  |
| CCS vs. HepG2 | 0.820 | 0.032 | 0.510 | 0.065 | -37.81 | <0.0001* |
| CCS vs. LX-2 | 0.820 | 0.032 | 0.909 | 0.047 | 10.82 | 0.2966 |
| HepG2 vs. LX-2 | 0.510 | 0.065 | 0.909 | 0.047 | 78.21 | <0.0001* |
| **96 h** |  |  |  |  |  |  |
| CCS vs. HepG2 | 0.811 | 0.037 | 0.510 | 0.047 | -37.13 | <0.0001* |
| CCS vs. LX-2 | 0.811 | 0.037 | 0.809 | 0.023 | -0.22 | 0.9995 |
| HepG2 vs. LX-2 | 0.510 | 0.047 | 0.809 | 0.023 | 58.71 | <0.0001* |

**Supplementary Table S15. Temporal changes in the MGV-derived spheroid compaction index within each culture model.** Mean MGV-derived compaction index ± SEM is presented for HepG2, LX-2, and CCS spheroids at 2, 24, 48, 72, and 96 h. Each subsequent time point was compared with the corresponding 2-h value. Relative change was calculated as [(MGV-derived compaction index at the comparison time − MGV-derived compaction index at 2 h)/MGV-derived compaction index at 2 h] × 100. Positive values indicate an increase, whereas negative values indicate a reduction in the MGV-derived compaction index relative to 2 h. Statistical analysis was performed using two-way ANOVA followed by Tukey’s multiple-comparisons test. p < 0.05 indicates statistical significance.

| **MGV-derived spheroid compaction index** | Mean 1 | SEM1 | Mean 2 | SEM2 | Relative change (%) | P-value |
| --- | --- | --- | --- | --- | --- | --- |
| **HepG2** |  |  |  |  |  |  |
| 2 h vs. 24 h | 0.356 | 0.091 | 0.514 | 0.034 | 44.67 | 0.17720 |
| 2 h vs. 48 h | 0.356 | 0.091 | 0.551 | 0.029 | 54.88 | 0.07880 |
| 2 h vs. 72 h | 0.356 | 0.091 | 0.510 | 0.065 | 43.40 | 0.19450 |
| 2 h vs. 96 h | 0.356 | 0.091 | 0.510 | 0.047 | 43.40 | 0.19480 |
| **LX-2** |  |  |  |  |  |  |
| 2 h vs. 24 h | 0.326 | 0.067 | 0.660 | 0.022 | 102.64 | <0.0001* |
| 2 h vs. 48 h | 0.326 | 0.067 | 0.823 | 0.036 | 152.81 | <0.0001* |
| 2 h vs. 72 h | 0.326 | 0.067 | 0.909 | 0.047 | 178.94 | <0.0001* |
| 2 h vs. 96 h | 0.326 | 0.067 | 0.809 | 0.023 | 148.42 | <0.0001* |
| **CCS** |  |  |  |  |  |  |
| 2 h vs. 24 h | 0.406 | 0.044 | 0.772 | 0.029 | 90.08 | <0.0001* |
| 2 h vs. 48 h | 0.406 | 0.044 | 0.840 | 0.029 | 106.77 | <0.0001* |
| 2 h vs. 72 h | 0.406 | 0.044 | 0.820 | 0.032 | 101.77 | <0.0001* |
| 2 h vs. 96 h | 0.406 | 0.044 | 0.811 | 0.037 | 99.58 | <0.0001* |

**Supplementary Table S16. Temporal changes in spheroid mean gray value (MGV) within each culture model.** Mean spheroid MGV ± SEM is presented for HepG2, LX-2, and CCS spheroids at 2, 24, 48, 72, and 96 h. Each subsequent time point was compared with the corresponding 2-h value. Relative change was calculated as [(MGV at the comparison time − MGV at 2 h)/MGV at 2 h] × 100. Positive values indicate an increase, whereas negative values indicate a reduction in spheroid MGV relative to 2 h. Statistical analysis was performed using two-way ANOVA followed by Tukey’s multiple-comparisons test. P < 0.05 indicates statistical significance.

| **MGV** | Mean 1 | SEM1 | Mean 2 | SEM2 | Relative change (%) | P-value |
| --- | --- | --- | --- | --- | --- | --- |
| **HepG2** |  |  |  |  |  |  |
| 2 h vs. 24 h | 1841.000 | 175.900 | 1536.000 | 66.250 | -16.57 | 0.17720 |
| 2 h vs. 48 h | 1841.000 | 175.900 | 1466.000 | 56.590 | -20.37 | 0.07880 |
| 2 h vs. 72 h | 1841.000 | 175.900 | 1544.000 | 125.600 | -16.13 | 0.19450 |
| 2 h vs. 96 h | 1841.000 | 175.900 | 1545.000 | 90.270 | -16.08 | 0.19480 |
| **LX-2** |  |  |  |  |  |  |
| 2 h vs. 24 h | 1773.000 | 40.910 | 1255.000 | 43.010 | -29.22 | <0.0001* |
| 2 h vs. 48 h | 1773.000 | 40.910 | 941.000 | 68.380 | -46.93 | <0.0001* |
| 2 h vs. 72 h | 1773.000 | 40.910 | 690.100 | 27.530 | -61.08 | <0.0001* |
| 2 h vs. 96 h | 1773.000 | 40.910 | 968.500 | 43.840 | -45.38 | <0.0001* |
| **CCS** |  |  |  |  |  |  |
| 2 h vs. 24 h | 1744.000 | 84.590 | 1039.000 | 56.480 | -40.42 | <0.0001* |
| 2 h vs. 48 h | 1744.000 | 84.590 | 908.900 | 54.880 | -47.88 | <0.0001* |
| 2 h vs. 72 h | 1744.000 | 84.590 | 947.900 | 60.700 | -45.65 | <0.0001* |
| 2 h vs. 96 h | 1744.000 | 84.590 | 965.000 | 70.960 | -44.67 | <0.0001* |

**Supplementary Table S17. Pairwise comparisons of spheroid mean gray values (MGV) among culture models.** Mean spheroid MGV ± SEM is presented for HepG2, LX-2, and CCS spheroids at 2, 24, 48, 72, and 96 h. Relative differences (%) were calculated as [(Mean 2 − Mean 1)/Mean 1] × 100, with the first model in each comparison used as the reference. Positive values indicate that Model 2 had a higher mean MGV than Model 1, whereas negative values indicate a lower mean MGV. Statistical analysis was performed using two-way ANOVA followed by Tukey’s multiple-comparisons test to compare culture models within each time point. P < 0.05 indicates statistical significance.

| **MGV** | Mean 1 | SEM1 | Mean 2 | SEM2 | Relative change (%) | P-value |
| --- | --- | --- | --- | --- | --- | --- |
| **2 h** |  |  |  |  |  |  |
| CCS vs. HepG2 | 1744.000 | 84.590 | 1841.000 | 175.900 | 5.56 | 0.7051 |
| CCS vs. LX-2 | 1744.000 | 84.590 | 1773.000 | 40.910 | 1.66 | 0.9567 |
| HepG2 vs. LX-2 | 1841.000 | 175.900 | 1773.000 | 40.910 | -3.69 | 0.8528 |
| **24 h** |  |  |  |  |  |  |
| CCS vs. HepG2 | 1039.000 | 56.480 | 1536.000 | 66.250 | 47.83 | <0.0001* |
| CCS vs. LX-2 | 1039.000 | 56.480 | 1255.000 | 43.010 | 20,79 | 0.0847 |
| HepG2 vs. LX-2 | 1536.000 | 66.250 | 1255.000 | 43.010 | -18.29 | 0.0176* |
| **48 h** |  |  |  |  |  |  |
| CCS vs. HepG2 | 908.900 | 54.880 | 1466.000 | 56.590 | 61.29 | <0.0001* |
| CCS vs. LX-2 | 908.900 | 54.880 | 941.000 | 68.380 | 3.53 | 0.9445 |
| HepG2 vs. LX-2 | 1466.000 | 56.590 | 941.000 | 68.380 | -35.81 | <0.0001* |
| **72 h** |  |  |  |  |  |  |
| CCS vs. HepG2 | 947.900 | 60.700 | 1544.000 | 125.600 | 62.89 | <0.0001* |
| CCS vs. LX-2 | 947.900 | 60.700 | 690.100 | 27.530 | -27.20 | 0.0427 |
| HepG2 vs. LX-2 | 1544.000 | 125.600 | 690.100 | 27.530 | -55.30 | <0.0001* |
| **96 h** |  |  |  |  |  |  |
| CCS vs. HepG2 | 965.000 | 70.960 | 1545.000 | 90.270 | 60.10 | <0.0001* |
| CCS vs. LX-2 | 965.000 | 70.960 | 968.500 | 43.840 | 0.36 | 0.9993 |
| HepG2 vs. LX-2 | 1545.000 | 90.270 | 968.500 | 43.840 | -37.31 | <0.0001* |
